# Prognostic and immune infiltrative biomarkers of CENPO in pan–cancer and its relationship with lung adenocarcinoma cell proliferation and metastasis

**DOI:** 10.1186/s12885-023-11233-2

**Published:** 2023-08-09

**Authors:** Yuanbiao Wang, Daowen Ye, Ying Li, Fenghong Lv, Wanbo Shen, Hui Li, Linghan Tian, Zongling Fan, Yanling Li, Yan wang, Feng Li, Yan Chen

**Affiliations:** 1 Department of Yunnan Tumor Research Institute, The Third Affiliated Hospital of Kunming Medical University, Yunnan Cancer Hospital, Kunming, 650118 China; 2 Ganzhou Cancer Hospital, Ganzhou, 341000 China; 3 Department of Hepatobiliary and Pancreatic Surgery, The Third Affiliated Hospital of Kunming Medical University, Yunnan Cancer Hospital, Kunming, 650118 China

## Abstract

**Background:**

The centromere protein O (CENPO) is an important member of the centromere protein family. However, the role of CENPO in pan–cancer and immune infiltration has not been reported. Here, we investigated the role of CENPO in pan–cancer and further validated its role in lung adenocarcinoma (LUAD) by in vitro experiments.

**Method:**

The UCSC Xena database and The Cancer Genome Atlas (TCGA)–LUAD data were used to assess the expression levels of CENPO. The potential value of CENPO as a diagnostic and prognostic biomarker for pan–cancer was evaluated using TCGA data and the GEPIA database. The -expression profiles of LUAD patients and the corresponding clinical data were downloaded for correlation analysis. The role of CENPO in immune infiltration was investigated using the UCSC Xena database. Subsequently, qRT–PCR was performed to detect the expression of CENPO. Cell proliferation, migration, and invasion were determined using CCK–8, wound–healing assay, and transwell assay, respectively.

**Results:**

CENPO is highly expressed in most cancers, and the upregulation of CENPO is associated with poor prognosis in many cancers. CENPO expression correlates with age, TNM stage, N stage, T stage, and receipt of radiotherapy in LUAD patients, and LUAD patients with high CENPO expression have poorer overall survival (OS) and disease–free survival (DFS). In addition, CENPO expression is associated with immune cell infiltration and immune checkpoint inhibitors. Moreover, the expression of CENPO was closely related to the expression of tumor mutational load and microsatellite instability. In vitro experiments showed that CENPO expression was increased in LUAD cell lines and that knockdown of CENPO significantly inhibited the proliferation, cell invasion, and migration ability of LUAD cells.

**Conclusion:**

CENPO may be a potential pan–cancer biomarker and oncogene, especially in LUAD. In addition, CENPO is associated with immune cell infiltration and may serve as a new molecular therapeutic target and effective prognostic marker for LUAD.

**Supplementary Information:**

The online version contains supplementary material available at 10.1186/s12885-023-11233-2.

## Introduction

Cancer is a leading cause of death, with 19.3 million new cancer cases and 10 million cancer deaths worldwide in 2020 [1]. The burden of cancer morbidity and mortality is rapidly escalating [2]. Despite the expanding number of treatments, including improvements in surgical modalities, immunotherapy, targeted therapies, and combination therapies [3], mortality from liver and lung cancers continues to increase yearly [4]. With the rapid development of next–generation sequencing, pan–cancer research has been widely used to identify tumor molecular markers and signaling pathways for a more comprehensive and in–depth understanding of the molecular mechanisms of tumor development [5–8]. Therefore, searching for novel diagnostic and prognostic biomarkers for cancer can help improve the prognosis of cancer patients.

Centromere protein O (CENPO), a member of the centromere protein (CENP) family, plays an important role in meiosis and mitosis, especially in chromosome segregation and normal cell division processes [9]. Many CENP family members have been identified, including CENPA, CENPL, CENPI and CENPU, and CENPO, which are associated with poor prognosis [6, 9–12]. CENPO–like proteins are associated with the prevention of premature sister chromatid separation and are associated with the cell cycle [13]. Recent studies have demonstrated that breast cancer patients with high CENPO expression have a poor prognosis and are independent factors affecting the distant recurrence–free survival (DRFS) of breast cancer patients [14]. High CENPO expression promotes the proliferation of gastric cancer cells and is positively associated with poor prognosis in gastric cancer [12]. CENPO is highly expressed in CRC (Colorectal Cancer) tissues and cells as positively associated with the malignancy of CRC degree and positively correlated with tumor progression possibly through EMT and PI3K/AKT signaling pathways [15]. CENPO upregulation promoted bladder cancer progression [16]. However, an investigation into the role of CENPO in pan–cancer is still lacking. We found that CENPO was significantly upregulated in multiple cancers and correlated with lung adenocarcinoma (LUAD) staging, overall survival (OS), and DFS. Thus, we chose LUAD to validate our bioinformatics results.

This study assessed CENPO expression in various human tumors using The Cancer Genome Atlas (TCGA) RNA–seq. The prognostic impact of aberrant CENPO expression on pan–cancer was investigated using the GEPIA database, and the impact of CENPO expression on the prognosis of LUAD patients was analyzed in conjunction with clinical data. Functional enrichment and pathway analysis were then performed, and correlations with immune cell infiltration, tumor mutational load (TMB), microsatellite instability (MSI), and gene methylation were investigated to understand further the biological mechanisms of CENPO in the pathogenesis of LUAD. Subsequently, bioinformatics results were validated by in vitro experiments. We preliminarily explored the potential mechanism of action of CENPO in LUAD, and CENPO could be used as an immunotherapeutic target and prognostic marker for pan–cancer, especially LUAD.

## Methods

### Material and methods

#### CENPO mRNA expression analysis

The UCSC Xena database GDC–TCGA data were used to analyze the differential mRNA expression of CENPO in pan–cancer and the relationship between CENPO, and cancer stage was analyzed by the “Stage plots” module of R. Lung adenocarcinoma RNA–seq sequencing profiles were obtained from the UCSC Xena database TCGA–LUAD data (517 LUAD cases and 58 normal cases), (https://xenabrowser.net/datepages/). We first mined the TCGA–LUAD dataset for differentially expressed genes (DEGs) in LUAD samples vs normal samples by the R limma package and then presented DEGs as a volcano map using Volcano map [17] (the criteria for determining differential gene expression were *P* < 0.05 and logFC absolute value > 1). Cases with insufficient or missing data were removed from subsequent data processing.

### Diagnostic value and prognostic analysis of CENPO

The diagnostic value of CENPO in pan–cancer was determined by plotting ROC curves from the GDC–TCGA data of the UCSC Xena database. AUC > 0.8 was thought as a high diagnostic value. In addition, the potential prognostic value of CENPO in pan–cancer was analyzed by overall survival (OS) and disease–free survival (DFS) from the GEPIA database (http://gepia.cancer-pku.cn/). The GEPIA database was divided into high and low expression groups based on the median CENPO expression in 33 cancers. KM survival curve analysis was performed based on the overall survival and disease-free survival of patients in the above groups, respectively, and risk ratios were calculated based on the Cox PH model.

### Methylation analysis and tumor mutation load

To screen methylation–regulated genes, we analyzed the correlation between CENPO expression and methylation in pan–cancer using GDC–TCGA data from the UCSC Xena database. Tumor mutational load (TMB) and microsatellite instability (MSI) analyses were performed on 33 tumors in the UCSC Xena database GDC–TCGA data via the “fmsb” package of R4.2.0.3.4. Next, the data were visualized using the radar chart package, and the obtained TMB correlation coefficients were all between 0.8 and -0.8, and the obtained MSI correlation coefficients were all between 0.4 and -0.4.

### Correlation of CENPO expression with clinical factors of LUAD and prognostic analysis

The correlation between CENPO expression and clinical characteristics, including gender, age, TNM stage, T stage, N stage, distant metastasis, and history of radiation therapy, were analyzed in 517 patients with LUAD in the TCGA database. To further evaluate the prognostic value of CENPO in patients with LUAD, univariate and multifactorial COX proportional risk regression analyses were performed to investigate the effects of CENPO and various clinical characteristics on OS in patients with LUAD, and HR and 95% confidence intervals were evaluated. In addition, we constructed column line plots based on CENPO expression values and pathological staging to facilitate the application of CENPO in the clinical prognostic assessment of LUAD. Finally, we assessed the predictive accuracy of the column line plots by calibration curves.

### Functional enrichment and immune infiltration analysis

Functional enrichment analysis of DEGs was performed by clusterProfiler in R software for Gene Ontology (GO) with the Kyoto Encyclopedia of Genes and Genomes (KEGG) [18, 19]. GO is commonly used to annotate genes and analyze their biological processes, which helps to reveal the functional mechanisms behind the patterns observed in transcriptomics, genomics, and proteomic**s** data, which are classified into cellular components (CC), molecular functions (MF), and biological processes (BP). Furthermore, KEGG is a reference knowledge base for the biological interpretation of large–scale molecular datasets. From this, key points of pathway mechanisms involved in differential genes in living systems can be obtained. Stromal cell score (StromalScore), immune cell score (ImmuneScore), and combined stromal and immune cell score (ESTIMATEScore) were performed by R4.2.0 for each tumor sample of TCGA–LUAD data in the UCSC Xena database, and the expression of CENPO was analyzed for its correlation with the tumor microenvironment correlation, immune cells, immune checkpoints.

### Drug sensitivity analysis

The list of cgp2016ExprRma drugs was obtained by the R package, CENPO was divided into high and low expression groups by median, and CENPO expression in LUAD was analyzed with a drug sensitivity analysis (IC50). For filtered conditions, the *P*–value was less than 0.001.

### Cell culture condition

The human normal lung epithelial cell line Beas–2b, LUAD cell lines XWLC–05, H838, A549, SPCA–1, and H1299 (Cell Bank of Chinese Academy of Sciences, Kunming, China) were placed in an incubator at 37 °C containing 5% CO_2_ using Roswell Park Memorial Institute (RPMI)– 1640 medium (GIBCO) and 10% fetal bovine serum (FBS, GIBCO).

### RNA interference and quantitative real–time PCR

GEMA chemically synthesized CENPO–specific small interfering RNAs (CENPO siRNAs), negative control siRNAs, and three RNA interference target sequences were designed (Table 1). Subsequently, cells were inoculated in 6–well plates and transfected when cell fusion reached approximately 50%. CENPO expression plasmids were transfected into cells using Lipofectamine 2000 (Invitrogen) transfection reagent according to the manufacturer's instructions. After 6 h of transfection, the cells were harvested 48 h later, and qRT–PCR verified the transfection efficiency. According to the kit instructions, RNA was extracted from A549 and H1299 cells 48 h after transfection using a Trizol reagent (Invitrogen, Carlsbad, CA, USA). An iScript cDNA Synthesis Kit (Bio–Rad, USA) was used to reverse transcribe the RNA to cDNA, followed by iTaq Universal SYBR (Bio–Rad, USA). Finally, iTaq Universal SYBR (Bio–Rad, USA) was used for qRT–PCR. The relative mRNA expression of CENPO was quantified using the cycling threshold (Ct) value and normalized using the 2^−ΔΔ^^Ct^ method. The primer sequences are summarized in Table 1. GAPDH was used as a reference control.

**Table 1:** The sequences of primers for qRT-PCR

primer	sequence
CENPO For	GGGAGGAAGTACCAGGCAGACC
CENPO Rev	CAATCTAGCACAGAACGGGAAGGAC
CENPO-Homo-1 For	GUGCUAUUGCUGUAUAATT
CENPO-Homo-1 Rev	UUAUACAGCAAUCUUAGCACTT
CENPO-Homo-2 For	CCGCAUACAUCACCAUUCATT
CENPO-Homo-2 Rev	UGAAUGGUGAUGUAUCCGGTT
CENPO-Homo-3 For	CCAGCGUGAAAGCAUGUAUTT
CENPO-Homo-3 Rev	AUACAUGCUUUCACGCUGGTT
GAPDH For	GGAGTCCACTGGCGTCTTCA
GAPDH Rev	GTCATGAGTCCTTCCACGATACC

### Cell counting kit–8 assay and colony formation assay

Cell proliferation was assessed using the CCK–8 assay. A549 and H1299 cells were digested after 48 h transfection and inoculated in 96–well plates at a density of 2,000 cells/well. After 1, 2, 3, 4, and 5 days of incubation, 10 μl CCK8 solution was added sequentially to each well, and the absorbance (OD) was measured at a wavelength of 450 nm using an enzyme marker after 2 h. The 5–day data were calculated, and cell proliferation curves were plotted. The experiment was repeated three times under the same conditions. A549 and H1299 cells were inoculated in 6–well plates at 500 cells/well and incubated in an incubator at 37 °C in humid air containing 5% CO_2_ for 14 days until the colonies were visible to the naked eye, followed by washing the colonies twice with PBS and staining with 0.25% crystal violet for 1 h. The colonies were counted and statistically analyzed using ImageJ software.

### Cell cycle test

Cells were collected 48 h after transfection and each tube contained 1 × 10^6^ cells. Using the cell cycle assay kit from biosharp, each sample was treated with 500 µl of staining buffer, 25 μl of propidium iodide staining solution (20 ×), and 10 µl of RNase A (50 ×) staining for 30 min. Immediately thereafter, the samples were assayed by flow cytometry.

### Wound–healing assay and Transwell invasion assay

The si–transfected (siNC, siCENPO) A549 and H1299 cells were inoculated in 6–well plates at a density of 1 × 10^6^ cells/well. The cells were placed under a microscope for image acquisition at 0 h, 24 h, and 48 h. First, the distance (μm) between scratches at different time points was quantified using Image J software (National Institutes of Health) to calculate the healing area (initial scratch width– final scratch width)/initial scratch width × 100%). Next, A549 and H1299 cells were placed in a Transwell chamber (24–well, 8 mm well) (Corning) at a density of 3 × 10^4^ cells/well and incubated for 24 h at 37 °C in a 5% CO_2_ incubator. The inner chamber contains 100 μl of cell suspension, and the outer chamber was 600 μl of 1640 medium containing 20% FBS. Afterward, the non–invasive cells in the upper chamber were removed Next, the cells attached to the polycarbonate membrane were fixed with 4% pre–cooled paraformaldehyde for 30 min and stained with 0.1% crystal violet for 20 min at room temperature. Afterward, the cells were placed under a 100 × microscope, and images were captured from five randomly selected fields of view.

### Statistical analysis

Data were analyzed and plotted using SPSS 25.0 (IBM, Armonk, NY, USA) and GraphPad Prism 8.0 software (GraphPad Software Inc., San Diego, CA, USA), and analyzed using R language. The results represent at least three replicate experiments and are expressed as Mean $\pm$ SD. Differences between two groups were compared using an unpaired t–test, and data from more than three groups were analyzed using one–way ANOVA. The Wilcoxon or Kruskal–Wallis test was employed to contrast the data from different groups. *P* < 0.05 was considered statistically significant (*P < 0.05; **P < 0.01; ***P < 0.001; ****P < 0.0001).

## Results

### CENPO is upregulated in multiple cancers

**Fig. 1:**
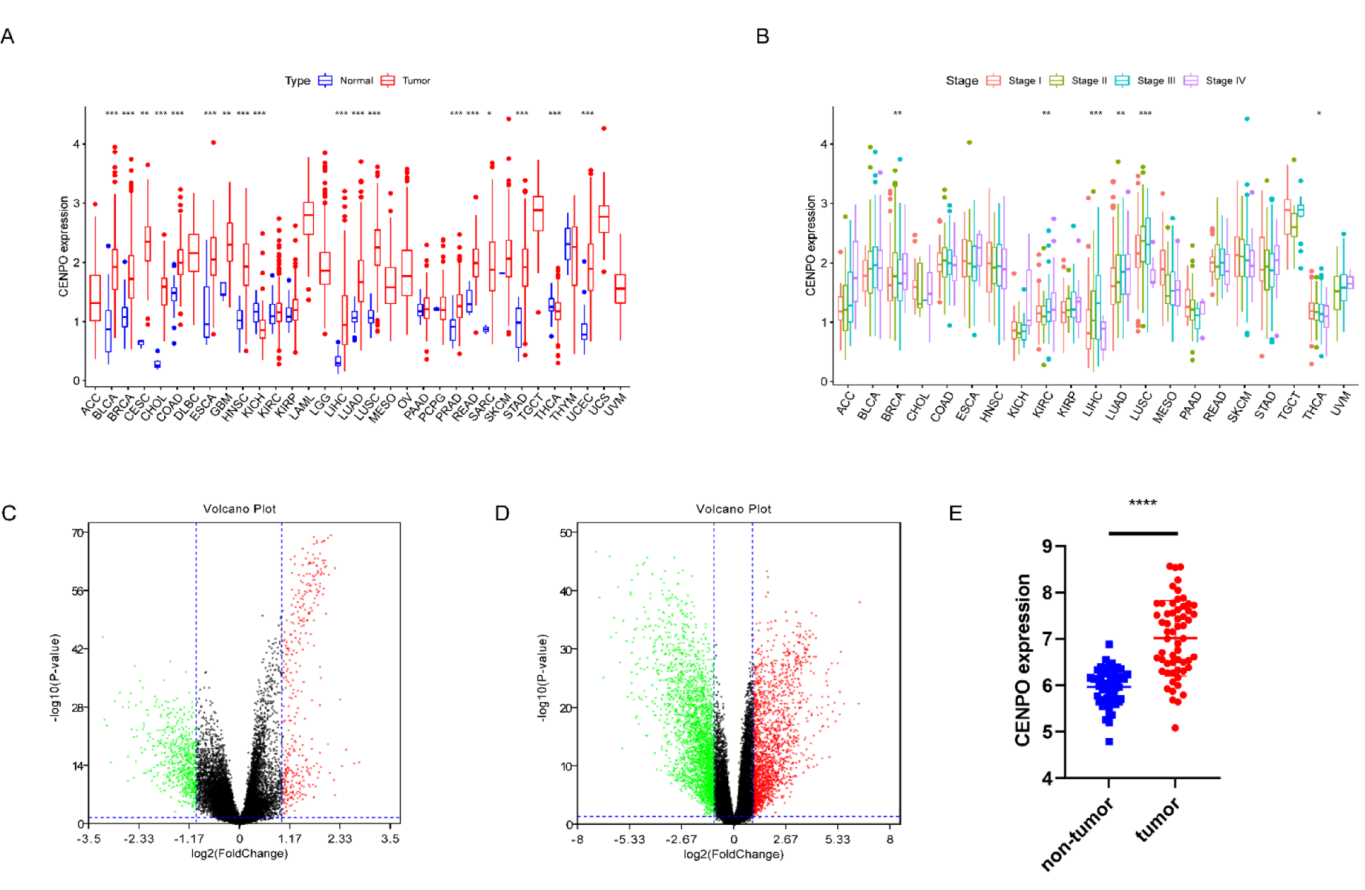
CENPO expression profile in pan–cancer. **A** CENPO expression in TIMER 2.0 database. **B** Correlation between CENPO expression and pan–cancer stage. **C** 960 DEGs were associated between LUAD and CENPO. **D** 58 pairs of paired LUAD tissue DEGs. **E** 58 pairs of paired LUAD tissues with CENPO differential expression

We analyzed CENPO expression in pan–cancer using the UCSC Xena database GDC–TCGA data. The results obtained indicate that CENPO is upregulated in most cancers (Fig. 1A), including urothelial carcinoma of the bladder (BLCA), breast cancer invasive carcinoma (BRCA), cervical cancer (CESC), bile duct cancer (CHOL), colon adenocarcinoma (COAD), esophageal cancer (ESCA), glioblastoma multiforme (GBM), head and neck squamous cell carcinoma (HNSC), hepatocellular carcinoma (LIHC), lung adenocarcinoma (LUAD), lung squamous cell carcinoma (LUSC), prostate adenocarcinoma (PRAD), rectal adenocarcinoma (READ), sarcomatoid lung cancer (SARC), gastric adenocarcinoma (STAD), and uterine somatic endometrial cancer (UCEC). However, CENPO was downregulated in renal cell carcinoma (KICH), and thyroid cancer (THCA) (Fig. 1A). In addition, CENPO expression is correlated with the staging of BRCA, KIRC, LIHC, LUAD, LUSC, and THCA (Fig. 1B). Based on the median expression of CENPO in 517 LUAD patients divided into high and low expression groups, differential analysis was performed to obtain 960 differential genes (DEGs) (Fig. 1C). Analysis of differential genes in 58 pairs of LUAD in paired samples with complete data of cancer and paracancerous tissue produced a volcano plot showing 4,220 DEGs (Fig. 1D). From the analysis of CENPO expression in 58 pairs of paired samples with complete data of cancer versus paraneoplastic tissues in LUAD, it could be concluded that the expression of CENPO was significantly higher in tumor tissues of LUAD patients than in normal tissues (Fig. 1E).

### Diagnostic and prognostic value of CENPO in pan–cancer

**Fig. 2:**
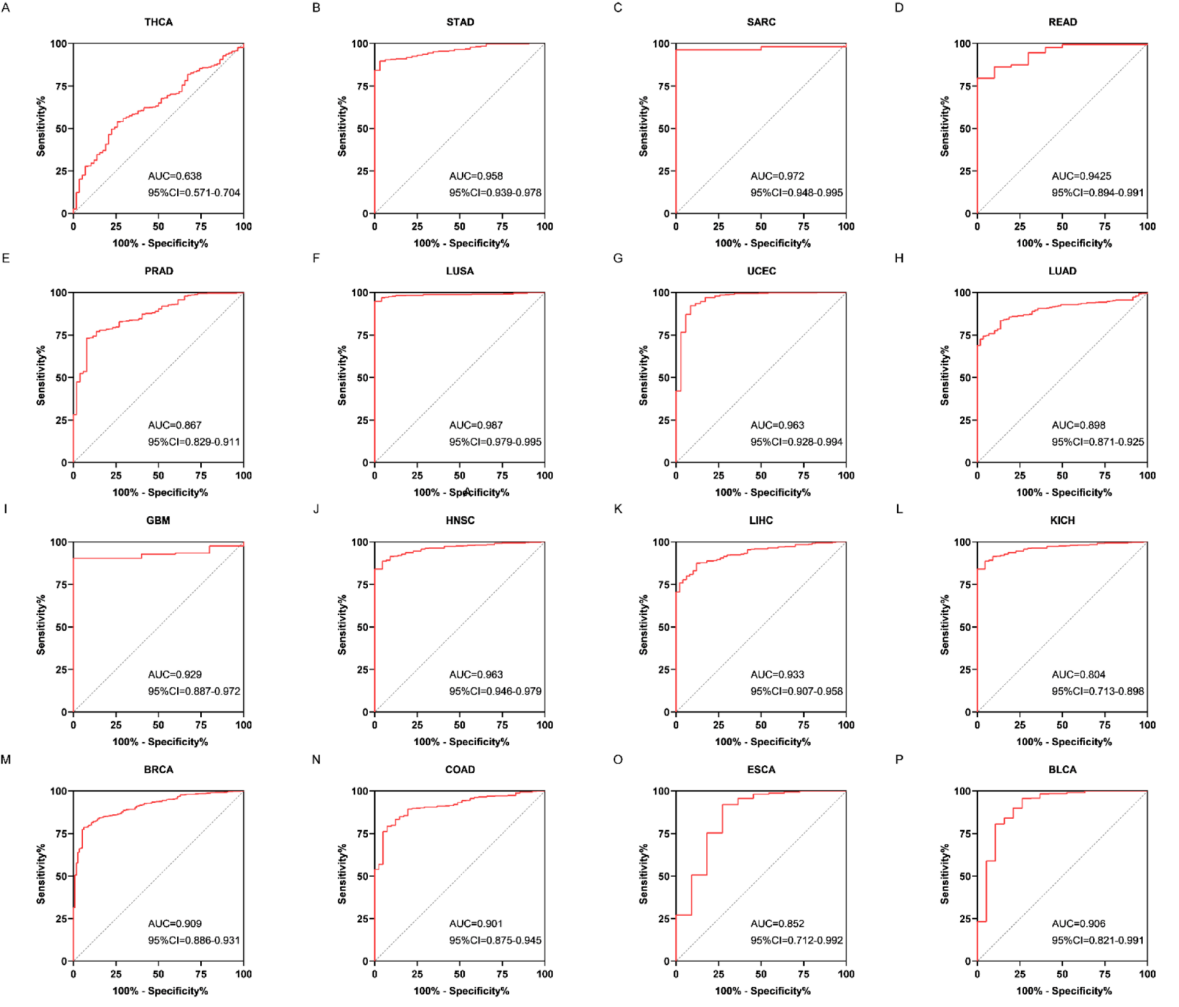
Diagnostic value of CENPO in pan–cancer analysis. **A**–**P** ROC curves of CENPL in THCA, STAD, SARC, READ, PRAD, LUSA, UCEC, LUAD, GBM, HNSC, LIHC, KICH, BRCA, COAD, ESCA, and BLCA

**Fig. 3:**
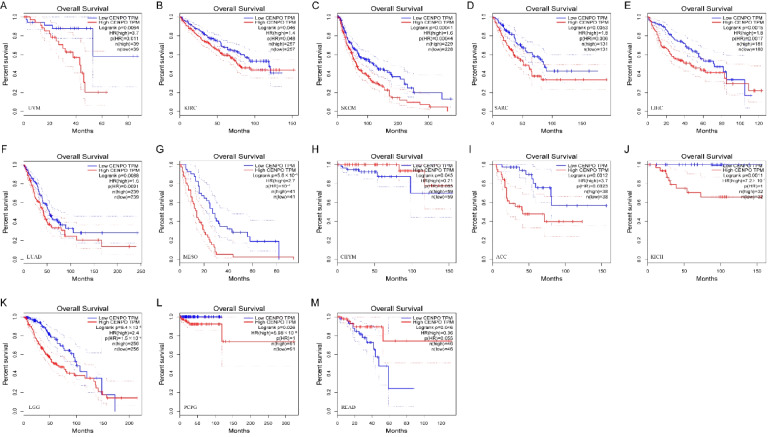
Prognostic value of CENPO in pan–cancer analysis (**A**–**M**) Kaplan–Meier analysis of the association between CENPO expression and OS. TPM: Transcripts Per Kilobase of exon model per Million mapped reads

**Fig. 4:**
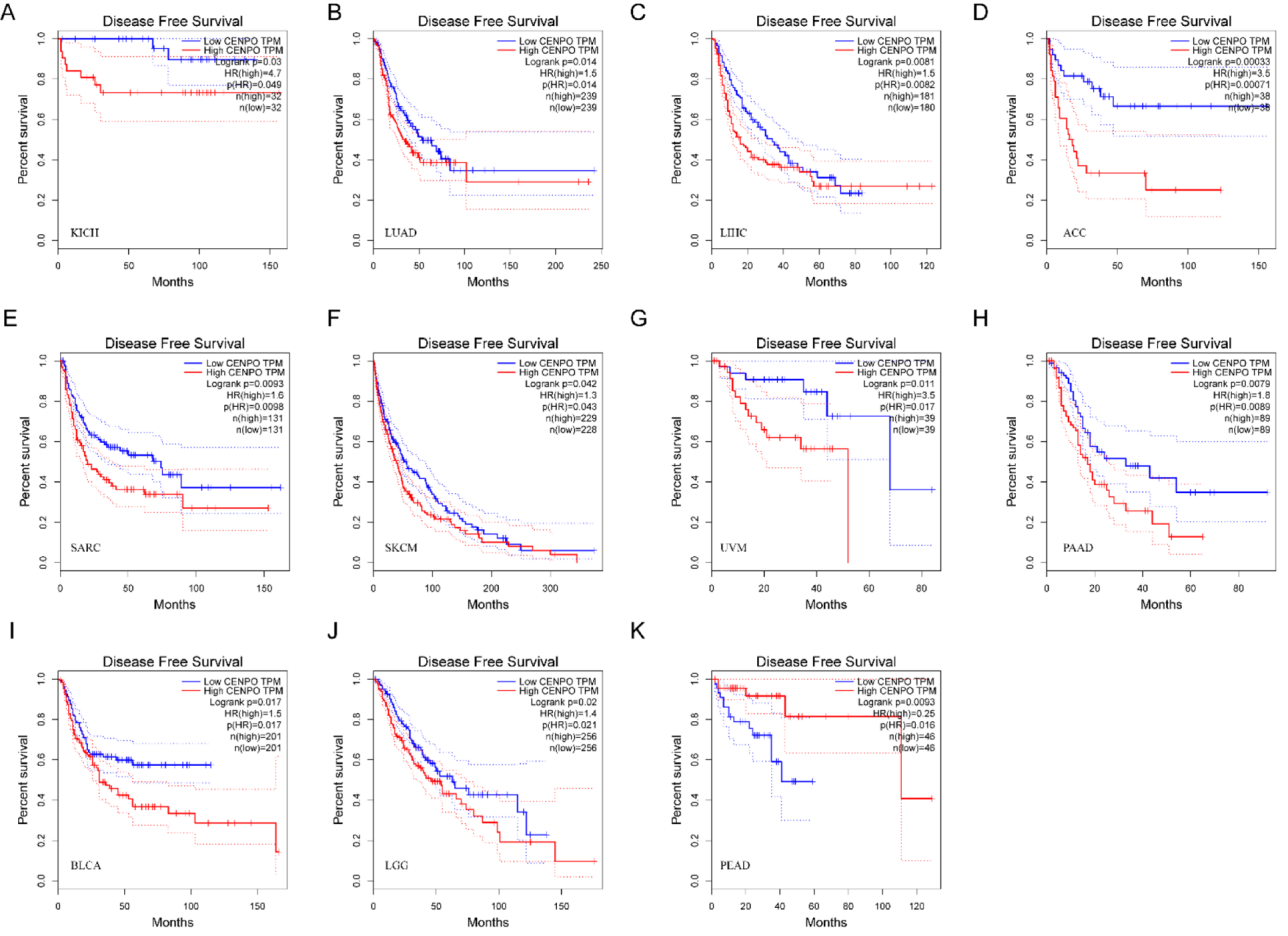
Prognostic value of CENPO in pan–cancer analysis. **A**–**K **Kaplan–Meier analysis of the association between CENPO expression and DFS

We analyzed the diagnostic value of CENPO in various cancers using ROC curves. ROC diagnostic curves were drawn based on the median CENPO expression of 33 cancers, which were divided into high and low expression groups. Figure 2(A–P) shows that CENPO had high diagnostic value in STAD, SARC, READ, LUSA, UCEC, LUAD, GBM, HNSC, LIHC, KICH, BRCA, COAD, ESCA, and BLCA (AUC: 0.8–1), and AUC = 0.638 in THCA, AUC = 0.704 in PRAD. These results suggest that CENPO has good diagnostic value for 14 cancer types and acceptable diagnostic value for THCA and PRAD. KM survival curves showed that increased expression of CENPO was linked to poor OS in UVM (*P* = 0.0064), KIRC (*P* = 0.046), SKCM (*P* = 0.00041), SARC (*P* = 0.0053), LIHC (*P* = 0. 0015), LUAD (*P* = 0.0088), MESO (*P* = 5.8e–05), ACC (*P* = 0.0012), KICH (*P* = 0.0011), LGG (*P* = 8.4e–06), and PCPG (*P* = 0.026) (Fig. 3A–L). In contrast, increased expression of CENPO was associated with better OS for CHYM (*P* = 0.045) and READ (*P* = 0.046; Fig. 3H, M). DFS results showed that increased expression of CENPO was associated with poor prognosis in KICH (*P* = 0.03), LUAD (*P* = 0.014), LIHC (*P* = 0.0081), ACC (*P* = 0.00033), SARC (*P* = 0. 0093), SKCM (*P* = 0.042), UVM (*P* = 0.011), PAAD (*P* = 0.0079), BLCA (*P* = 0.017), and LGG (*P* = 0.02; Fig. 4A–J). In contrast, increased expression of CENPO was associated with better DFS in PEAD (*P* = 0.0093) (Fig. 4K). We downloaded 33 pan-cancer data from the TCGA database and performed CENPO differential expression analysis and survival analysis, taking the intersection of cancers with differential CENPO expression and cancers with significantly associated OS to obtain four cancers, KICH, LIHC, READ, and SARC. The clinicopathological characteristics of these four cancers and the expression of CENPO were then subjected to COX regression analysis, which showed that CENPO and Stage were independent risk factors for KICH. CENPO, age, and M were independent risk factors for LIHC. CENPO, age, and N stage were independent risk factors for READ. CENPO and age were independent risk factors for SARC (Supplementary Fig. 1).

### Methylation analysis and tumor mutation load

**Fig. 5:**
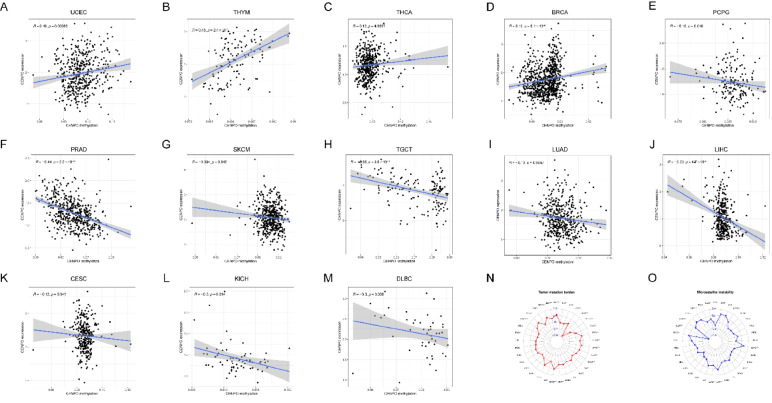
Methylation analysis and tumor mutation load. Correlation analysis of CENPO with methylation in pan–cancer. **A**–**D** CENPO expression is positively correlated with methylation. **E**–**M** CENPO expression is negatively correlated with methylation. Correlation of CENPO expression with TMB (**N**). Correlation of CENPO expression with MSI (O)

Methylation is a novel programmed inflammatory cell death type closely associated with tumor development [20]. To further investigate the mechanisms underlying the aberrant expression of CENPO in various cancers, we analyzed the relationship between CENPO expression and genomic methylation. The results showed that CENPO was positively associated with methylation in UCEC, THYM, THCA, and BRCA (Fig. 5A–D). Conversely, CENPO was negatively correlated with methylation in PCPG, PRAD, SKCM, TGCT, LUAD, LIHC, CESC, KICH, and DLBC (Fig. 5E–M). These findings suggest that methylation changes may affect the expression of CENPO in pan–cancer. Tumor mutational load (TMB) and microsatellite instability (MSI) influence tumorigenesis and progression. Therefore, the correlation between CENPO expression and TMB or MSI in 33 common cancer types was analyzed. CENPO levels were significantly correlated with TMB in ACC, UCES, THYM, THCA, STAD, SKCM, SARC, READ, PRAD, PAAD, LUSC, LUAD, LGG, COAD, CHOL, BRCA and BLCA (Fig. 5N). On the other hand, UVM, UCEC, SARC, LUSC, LUAD, LIHC, KIRP, ESCA, DLBC, COAD, CESC, BLCA, and CENPO were significantly correlated with MSI (Fig. 5O).

### Analysis of the clinical correlation of CENPO in LUAD

**Fig. 6:**
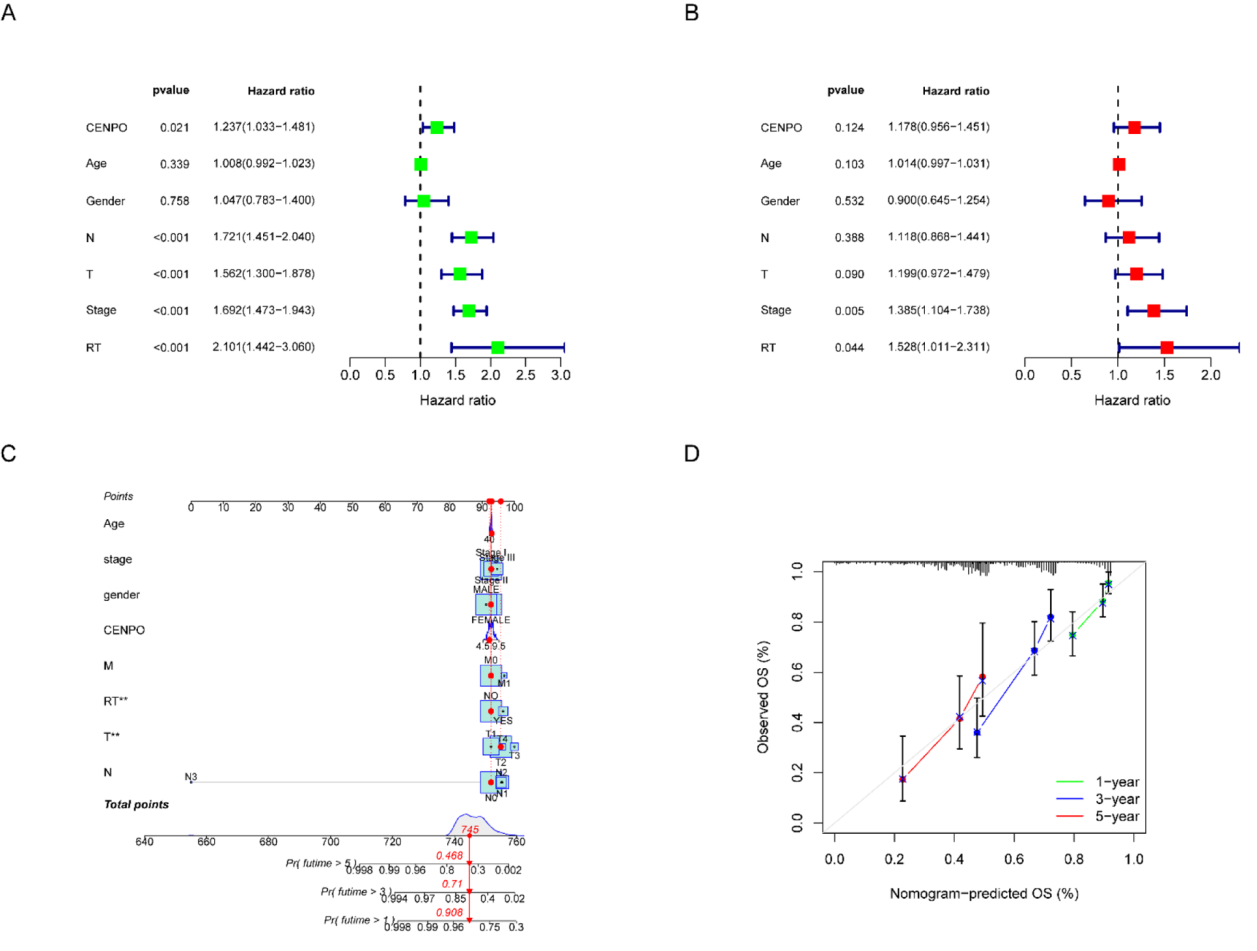
Prognostic analysis of CENPO in LUAD (**A**–**B**) The independent prognostic significance of CENPO in LUAD was analyzed by univariate and multivariate COX regression. **C** Columnar line graphs based on CENPO expression and pathological staging. **D** Corrected analysis plot of column line graphs

The correlation between CENPO expression and clinical characteristics of 517 LUAD patients in the TCGA database was analyzed. CENPO expression was correlated with gender, age, T stage, N stage, TNM stage, and whether or not radiotherapy was administered, and not with recurrence or distant metastasis (Table 2). These results suggest that CENPO expression may be related to the malignant pathological progression of LUAD. Meanwhile, to further evaluate the prognostic value of CENPO in LUAD, univariate and multifactorial COX regression, and ROC curve analyses were performed. COX regression analysis showed that CENPO expression was associated with an increased risk of death in patients with LUAD (*P* = 0.021, HR:1.237, 95% CI 1.033–1.481) (Fig. 6A), and multifactorial COX regression analysis showed that TNM stage and the presence or absence of radiation therapy were independent risk factors (*P* < 0.05) (Fig. 6B). In addition, to facilitate the application of CENPO in clinical evaluation, we constructed column line graphs (Fig. 6C) based on the expression and pathological stage of CENPO. Finally, we evaluated the accuracy of the prognostic assessment model for LUAD patients after 1, 3, and 5 years by calibration curves (Fig. 6D). The column line graph had relatively good accuracy.

**Table 2:** Correlation of clinicopathological features with CENPO expression in LUAD

Clinical Variable	n	CENPO		χ ^2^	*P*-value
		Low	High		
Gender					
Male	240	103	137	8.746	0.03*
Female	277	155	122		
Age(years)					
< 60	155	66	89	4.748	0.029*
≥ 60	362	192	170		
TNM stage				13.963	0.003**
I	277	154	123		
II	122	60	62		
III	84	29	55		
IV	26	9	17		
N stage					
N0	333	180	153	16.038	0.001**
N1	96	41	76		
N2	74	39	52		
N3	2	1	1		
M stage					
M0	347	170	177	0.117	0.733
M1	166	84	82		
T stage					
T1	170	102	68	12.119	0.007**
T2	278	121	157		
T3	47	22	25		
T4	19	11	8		
Recurrence					
Yes	151	61	90	3.602	0.058
No	243	122	121		
Radiation therapy				6.303	0.012*
Yes	60	21	39		
No	399	209	190		

^*^P < 0.05, **P < 0.01

Abbreviations: *LUAD* Lung adenocarcinoma, *TNM* Tumor node metastasis classification

### Functional enrichment and tumor microenvironment

**Fig. 7:**
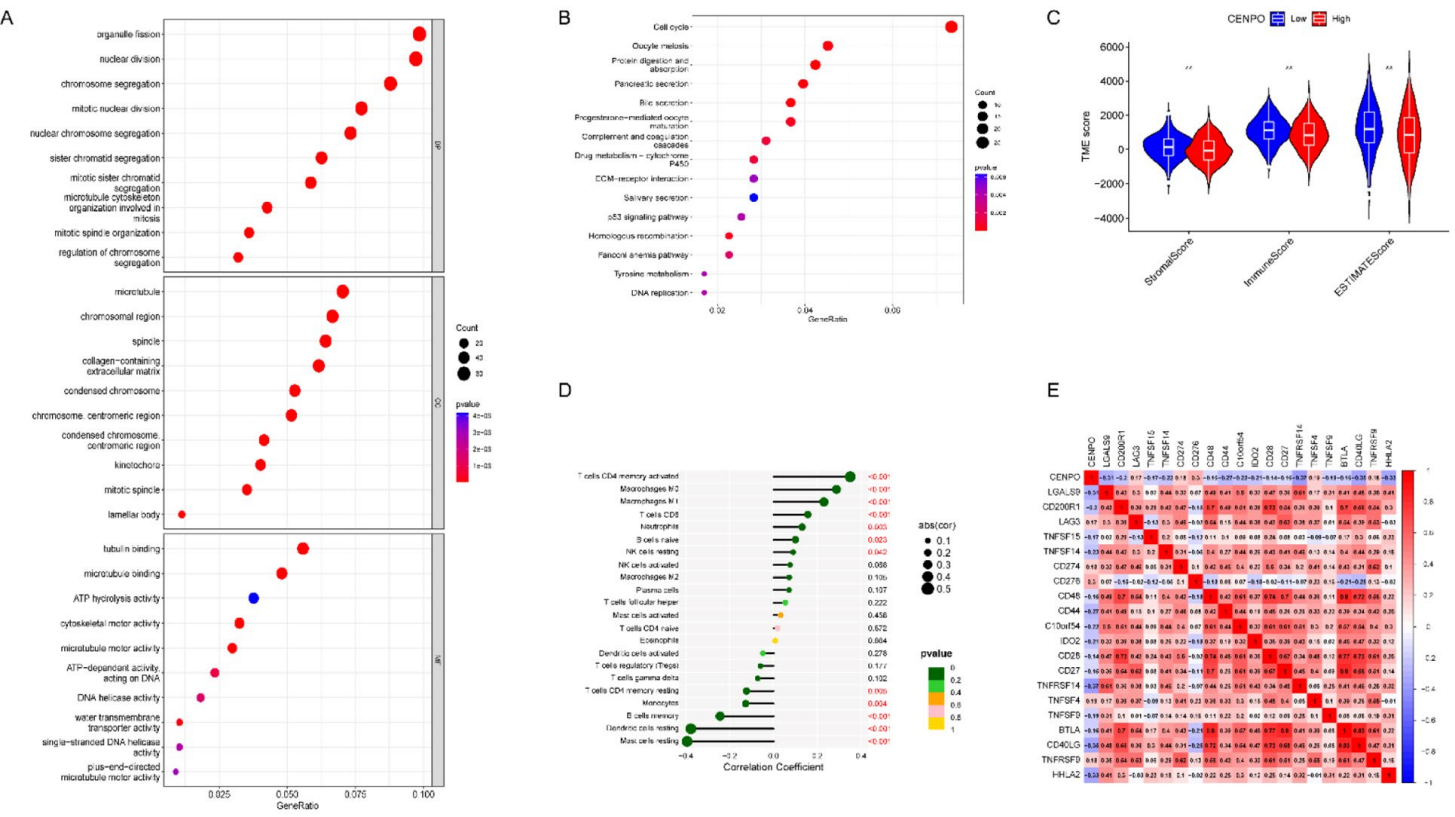
Functional enrichment and immune infiltration analysis. **A**–**B** GO and KEGG functional enrichment analysis of the molecules interacted with CENPO. **C** Correlation analysis of CENPO high and low expression groups with TME scores. **D** Correlation of CENPO expression with immune cell infiltration. **E** Correlation analysis of CENPO expression with immune checkpoint inhibitors

To further explore the function of CENPO in LUAD, we performed GO and KEGG functional enrichment analysis, as shown in Supplementary Table 2, yielding 970 GO items (766 BP items, 90 CC items, 114 MF items) and 35 KEGG pathways. The top 10 items under each classification are included in the bubble plots (Fig. 7A, B). GO analysis showed that these molecules were mainly involved in organelle fission, nuclear division, chromosome segregation, mitotic nuclear division, nuclear chromosome segregation, etc. Cellular component enrichment showed that these molecules were mainly enriched in microtubule, chromosomal region, spindle, and collagen–containing extracellular matrix, etc. Molecular functional enrichment analysis showed that these molecules were mainly associated with tubulin binding, microtubule binding, ATP hydrolysis activity, cytoskeletal motor activity, and microtubule motor activity. KEGG analysis showed that CENPO was associated with Cell cycle, Oocyte meiosis, protein digestion and absorption, Pancreatic secretion, Bile secretion, Progesterone–mediated oocyte maturation, Complement and coagulation cascades, Drug metabolism–cytochrome P450, ECM–receptor interaction, Salivary secretion, p53 signaling pathway, Homologous recombination, Fanconi anemia and other signaling pathways. Tumor microenvironment score (TME score) showed that StromalScore, ImmuneScore, and ESTIMATEScore were higher in the CENPO low expression group than in the CENPO high expression group (Fig. 7C). The results of correlation analysis between CENPO expression and immune cells showed that CENPO in LUAD was significantly and positively correlated with memory–activated CD4 T cells, Macrophages M0, Macrophages M1, T cells CD8, Neutrophils, B cells naive, and resting NK cells. Significant negative correlations were found with Mast cells resting, Dendritic cells resting, B cells memory, Monocytes, and memory resting CD4 T cells (Fig. 7D). In addition, we evaluated the correlation between CENPO expression and immune checkpoints, and showed that CENPO was significantly positively correlated with immune checkpoints such as CD276, CD274, TNFSF4, TNFRSF9, and LAG3. Meanwhile, CENPO was negatively correlated with immune checkpoints such as LGALS9, TNFRSF14, CD40LG, and HHLA2 (Fig. 7E).

### Drug sensitivity analysis

**Fig. 8:**
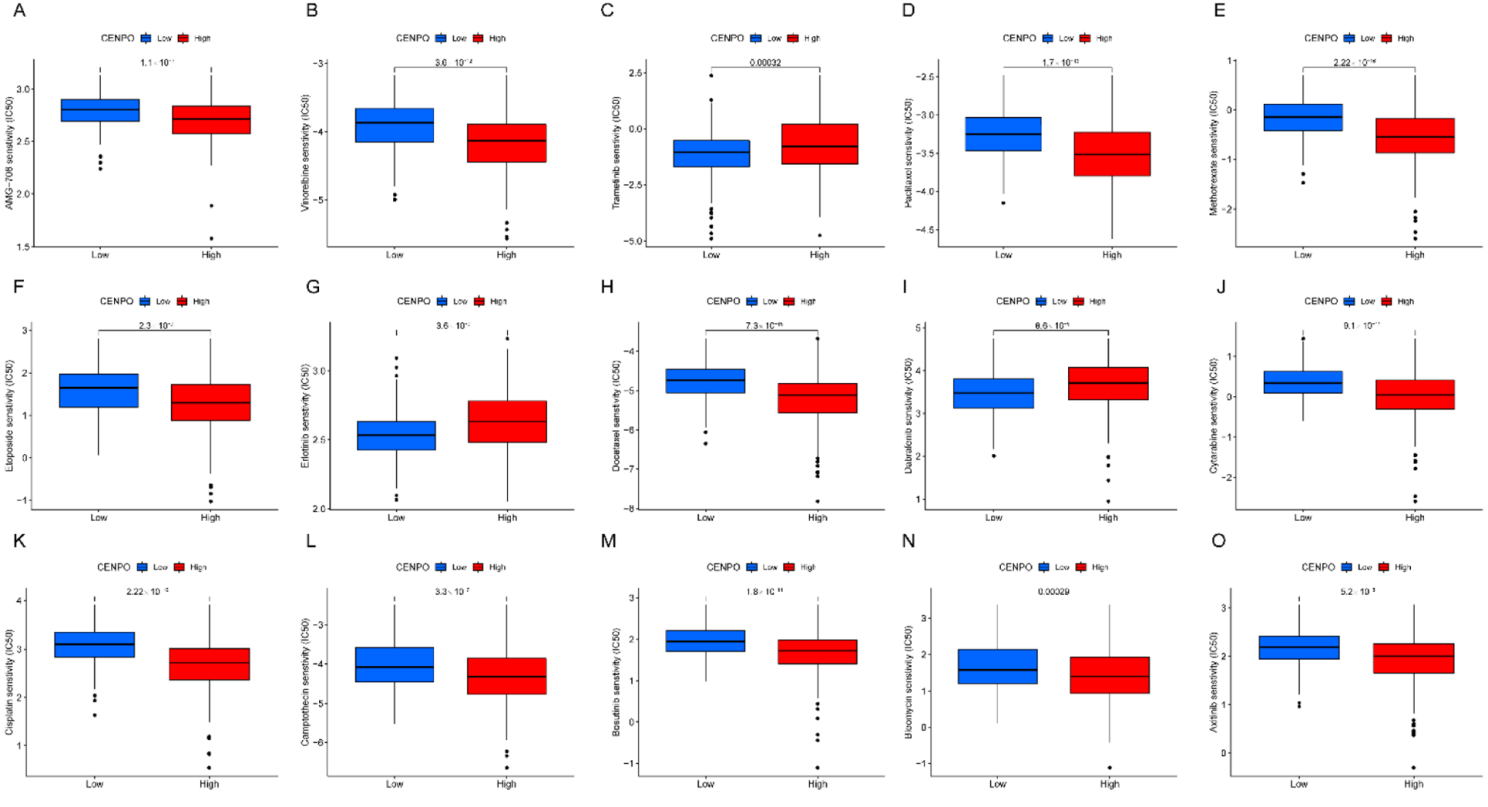
Drug sensitivity analysis. **A**–**O** CENPO expression in LUAD with drug sensitivity analysis (IC50)

One of the challenges of precision medicine is identifying biomarkers that predict cancer cells' response to drug therapy. Large–scale cancer pharmacogenomic studies offer the possibility to develop new markers [21]. Our screening results showed that the IC50 of 114 drugs correlated with the expression of CENPO (*P* < 0.001), 15 of which were clinically used lung cancer therapeutics (Fig. 8A–O). These data suggest that CENPO can be used as a biomarker for predicting LUAD drug therapy.

### CENPO knockdown inhibits LUAD cell proliferation

**Fig. 9:**
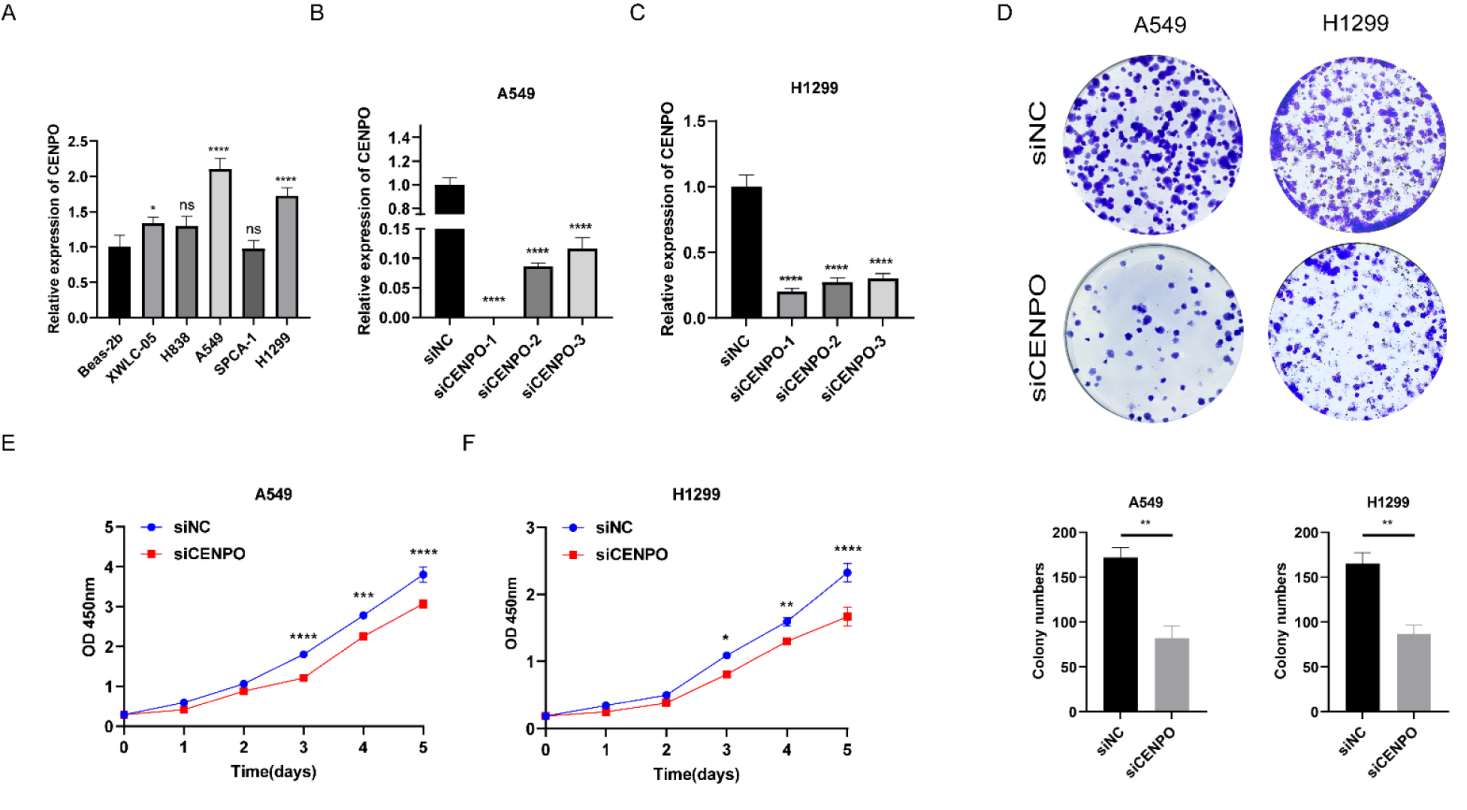
Validation of CENPO expression in LUAD. **A** CENPO mRNA expression in LUAD cell lines and normal lung epithelial cells. **B**–**C** Knockdown efficiency of CENPO mRNA in A549 and H1299 cells. **D** Clone formation assay. **E**–**F** CCK–8 assay to detect the proliferation of A549 and H1299 cells

To investigate the cellular function of CENPO in LUAD, we examined the mRNA expression levels of CENPO in lung normal epithelial cells BEAS–2b and five LUAD cell lines XWLC–05**,** H838, A549, SPCA–1**,** and H1299 by qRT–PCR. CENPO was significantly upregulated in the LUAD cell lines (Fig. 9A). A549 and H1299 cell lines were selected for CENPO knockdown for functional studies. Three siRNAs targeting CENPO were used for the knockdown, and the most efficient siRNA (CENPO–1) was selected for the next experiments (Fig. 9B, C). We investigated the effect of CENPO knockdown on cell proliferation by CCK–8 and clone formation assays. We showed that the downregulation of CENPO significantly reduced the proliferation viability of A549 and H1299 cells (Fig. 9E, F) and the number of colonies formed by the cells (Fig. 9D).

### Downregulation of CENPO inhibits the migration and invasion of LUAD cells

**Fig. 10:**
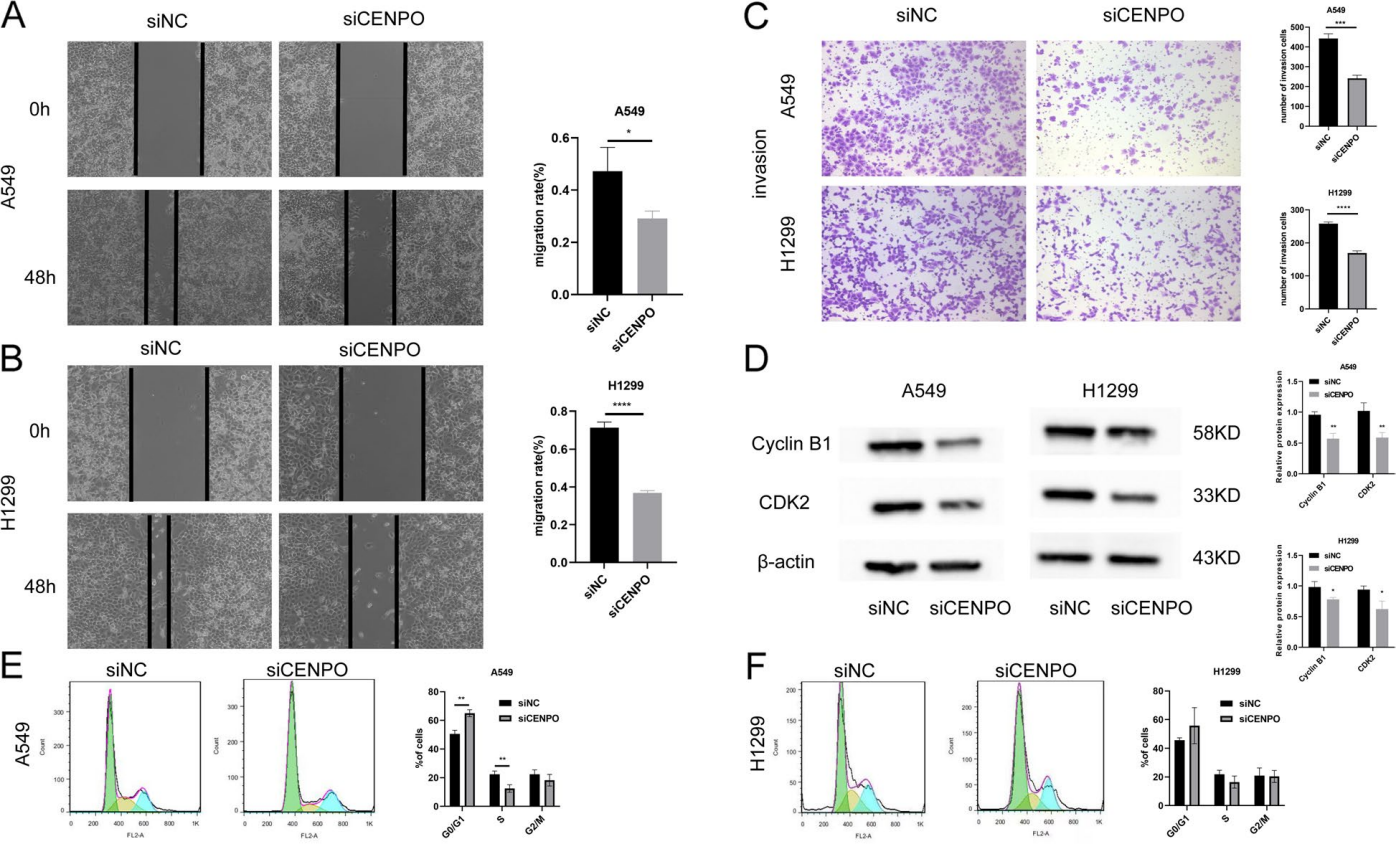
Effect of CENPO knockdown on the invasion and migration of A549 and H1299 cells. **A**–**C** The effect of CENPO knockdown on migration and invasion of A549 and H1299 cells was assessed using a wound–healing assay and transwell assay. **D** Western blot detected the expression of CDK2 and CyclinB1 in A549 and H1299 cells. **E**–**F **Cell cycle distribution was analyzed by Propidium iodide staining 48 h after A549 and H1299 cells were transfected with Si-CENPA or CENPA plasmids.The results represent experiments that were repeated at least three times. The results represent experiments that were repeated at least three times. Data are expressed as mean ± standard deviation

After cell scratching experiments, we found that the healing ability of A549 and H1299 cells was significantly reduced after the knockdown of CENPO (Fig. 10A, B). Furthermore, the Transwell invasion assay was also performed, and the results showed that the number of H1299 and A549 cells invaded was significantly reduced after the knockdown of CENPO (Fig. 10C). Based on the above results, we inferred that the knockdown of CENPO could inhibit migration and invasion of LUAD cell lines A549 and H1299. Furthermore, WB results showed that the knockdown of CENPO resulted in decreased expression of CDK2 and CyclinB1 in A549 and H1299 cells (Fig. 10D). Flow cytometry results showed that H1299 exhibited a consistent trend of change with A549 cells, although there was no statistically significant difference. It indicates that the knockdown of CENPO significantly induced G0/G1 phase arrest in A549 and H1299 cells (Fig. 10E–F).

## Discussion

Mitotic granules are an important component of chromosome segregation during meiosis and mitosis in normal cells. Their abnormal function can lead to mitotic arrest and altered chromosome numbers [22]. It has been reported that Centromere proteins control chromosome segregation and that abnormalities in Centromere proteins may lead to chromosomal instability in tumors, which in turn lead to tumor progression and drug resistance [23]. CENPO is associated with the development and progress of various tumors, including LUAD. These data suggest the potential of CENPO as a prognostic biomarker.

In this study, we verified that CENPO was significantly highly expressed in most cancer tissues and correlated with the TNM stage by the TCGA database. ROC curve analysis indicated that CENPO might serve as a pan–cancer diagnostic biomarker. Meanwhile, KM survival analysis showed that increased expression of CENPO was associated with poorer OS of UVM, KIRC, SKCM, SARC, LIHC, LUAD, MESO, ACC, KICH, LGG, and PCPG, whereas the increased expression of CENPO was associated with KICH, LUAD, LUSC, SARC, SKCM, UVM, ACC, BLCA LGG, LIHC, and PAAD poorer DFS. These findings suggest that CENPO may play a significant role in tumorigenesis and prognosis, and that abnormal CENPO may be a potential prognostic predictive molecule for cancer. Further analysis revealed that CENPO expression in LUAD was significantly correlated with gender, TNM stage, N stage, T stage, and whether radiotherapy was administered. Columnar plots were constructed to further evaluate the role of CENPO in LUAD prognosis.

Epigenetic mechanisms regulate the expression of immunosynaptic proteins by tumor cells, thereby suppressing immune function [24]. DNA methylation is a key epigenetic mechanism of immune regulation, and cancer cells often use epigenetic dysregulation to silence tumor suppressors or activate oncogenes [25]. Our results showed that CENPO expression was negatively correlated with methylation in LUAD, suggesting that CENPO may be involved in epigenetic modifications to regulate mitophagy assembly and mitophagy function [26]. The tumor microenvironment is a complex survival environment for tumor cells, mainly characterized by immune cells, mesenchymal cells, blood vessels, and an extracellular matrix [27]. In recent years, there has been increasing evidence that the tumor microenvironment and the level of immune cell infiltration are closely related to the growth and progression of tumor cells [28–31]. Although previous studies have demonstrated the prognostic importance of CENPO in various types of cancer, there are few reports on whether aberrant CENPO expression influences immune cell infiltration. Immune cells of the tumor microenvironment are associated with the efficacy of immunotherapy, including CD4 + T cells [32] CD8 + T cells [33], NK cells [34], and macrophage cells [35]. Our results show for the first time that CENPO expression in LUAD is significant positive associated with the infiltration of memory–activated CD4 T cells, M0 macrophages, M1 macrophages, T cells CD8, Neutrophils, naive B cells, and NK cell resting; our results also show a significant negative correlation with the resting Mast cells, resting Dendritic cells, memory B cells, Monocytes, and memory resting CD4 T cells. Macrophages have been reported to promote tumor progression through tissue remodeling and angiogenesis [36]. High infiltration of M1 macrophages significantly reduced survival time in prostate cancer patients [29]. The M1 macrophage content was significantly higher in the metastatic group than in the non–metastatic group of medulloblastoma [37]. Our results also showed that increased CENPO expression was associated with poor LUAD survival.

The level of immune cell infiltration in TME is critical for tumor prognosis [38]. An immune–based assay called “Immunoscore” was defined to quantify T–cell infiltration in situ and proved superior to the TNM classification of colorectal cancer patients [39]. Furthermore, ImmuneScore has been reported to predict that survival in CRC patients is superior to microsatellite instability [40]. Our results showed that StromalScore, ImmuneScore, and ESTIMATEScore were significantly lower in the CENPO high expression group, which may indicate that increased CENPO expression is associated with disturbed immune status in LUAD patients. Immune checkpoints are involved in physiological immune responses, and immune checkpoints and their ligands are upregulated in the tumor microenvironment of various malignancies to induce antitumor immune responses [41]. The clinical application of immune checkpoint inhibitors has improved the prognosis of many patients with advanced tumors [42]; we showed that CENPO was positively correlated with immune checkpoint CD276, TNFSF4, TNFSF9, CD247, and LAG3. Furthermore, it was negatively correlated with immune checkpoints LGALS9, CD44, TNFRSF14, CD40LG, and HHLA2. These results suggest that CENPO plays an important immunomodulatory role and may serve as a potential immunotherapeutic relevant biomarker. Previous studies reported that the CENP family is associated with immune function [6, 43], and our bioinformatics results also demonstrated that CENPO is associated with immune regulation. We then demonstrated by in vitro experiments that CENPO was highly expressed in A549 and H1299 cell lines and that the knockdown of CENPO significantly inhibited the proliferative activity, migration, and invasive ability of A549 and H1299 cells, suggesting that CENPO may affect LUAD metastasis. Studies have reported that the knockdown of CENPO inhibited GC cell growth, induced apoptosis, and decreased the expression of ATM, cyclin D1, and c–Jun [12]. Knockdown of CENPO in CRC downregulated the expression of N–cadherin, Vimentin, Snail, and CCND1, inhibited cell proliferation, and attenuated migration and invasion [15]. This is consistent with our Bioinformatics Analysis, the functional enrichment results showed significant enrichment of cell cycle, P53 pathway, and extracellular matrix. Thus, our study suggests that the knockdown of CENPO significantly decrease the expression of CDK2 and CyclinB1, and induce G0/G1 arrest in A549 and H1299 cells. We speculate that CENPO may promote LUAD progression by regulating the cell cycle. These results suggest that CENPO may be a potential molecular target for anti–tumor therapy in LUAD, and the specific mechanism of CENPO in regulating the development of LUAD will be further investigated in the future.

### Supplementary Information

**Additional file 1.**

**Additional file 2.**

**Additional file 3.**

**Additional file 4.**

**Additional file 5.**

**Additional file 6.**

**Additional file 7.**

**Additional file 8.**

**Additional file 9.**

**Additional file 10.**

**Additional file 11.**

**Additional file 12.**

**Additional file 13.**

**Additional file 14.**

**Additional file 15.**

**Additional file 16.**

**Additional file 17.**

## Data Availability

All data generated or analyzed during this study are included in this published article (and its Supplementary Information files). The datasets generated during and/or analyzed during the current study are available in the UCSC Xena(https://xenabrowser.net/datepages/) and GEPIA repository GEPIA (Gene Expression Profiling Interactive Analysis) (cancer-pku.cn).

## Abbreviations

CENPO: Centromere protein O
CRC: Colorectal Cancer
DEG: Differentially expressed genes
DFS: Disease–Free Survival
GEO: Gene Expression Omnibus
GO: Gene Ontology
GSEA: Gene Set Enrichment Analysis
KEGG: Kyoto Encyclopedia of Genes and Genomes
LUAD: Lung adenocarcinoma
MSI: Microsatellite instability
OS: Overall survival
TNM: Tumor node metastasis classification
TME: Tumor microenvironment
TMB: Tumor mutation burden
TCGA: The Cancer Genome Atlas
