# Supplementary figures and images for: Prognostic and immune infiltrative biomarkers of CENPO in pan–cancer and its relationship with lung adenocarcinoma cell proliferation and metastasis
Source: BMC Cancer. 2023 Aug 9;23:735. doi: 10.1186/s12885-023-11233-2 (PMC10410993; doi:10.1186/s12885-023-11233-2)

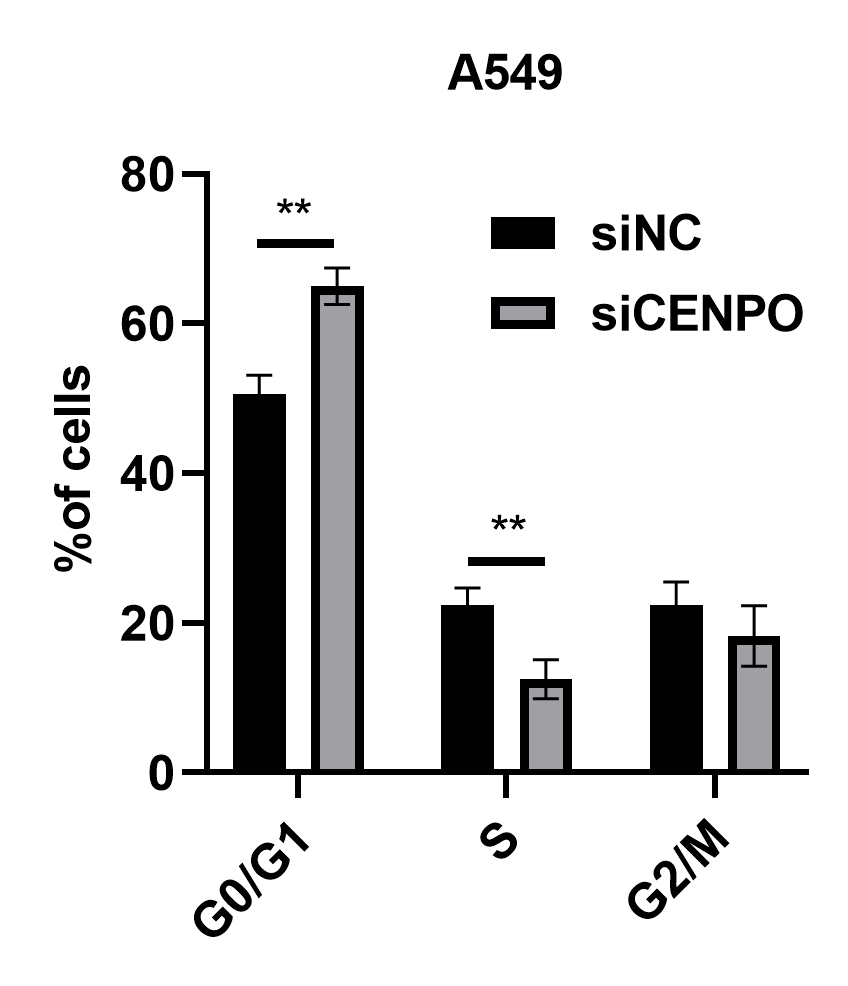

Supplement: Supplementary file 3 — Additional file 3. [file 12885_2023_11233_MOESM3_ESM.zip › A549 Cell cycle.tif]

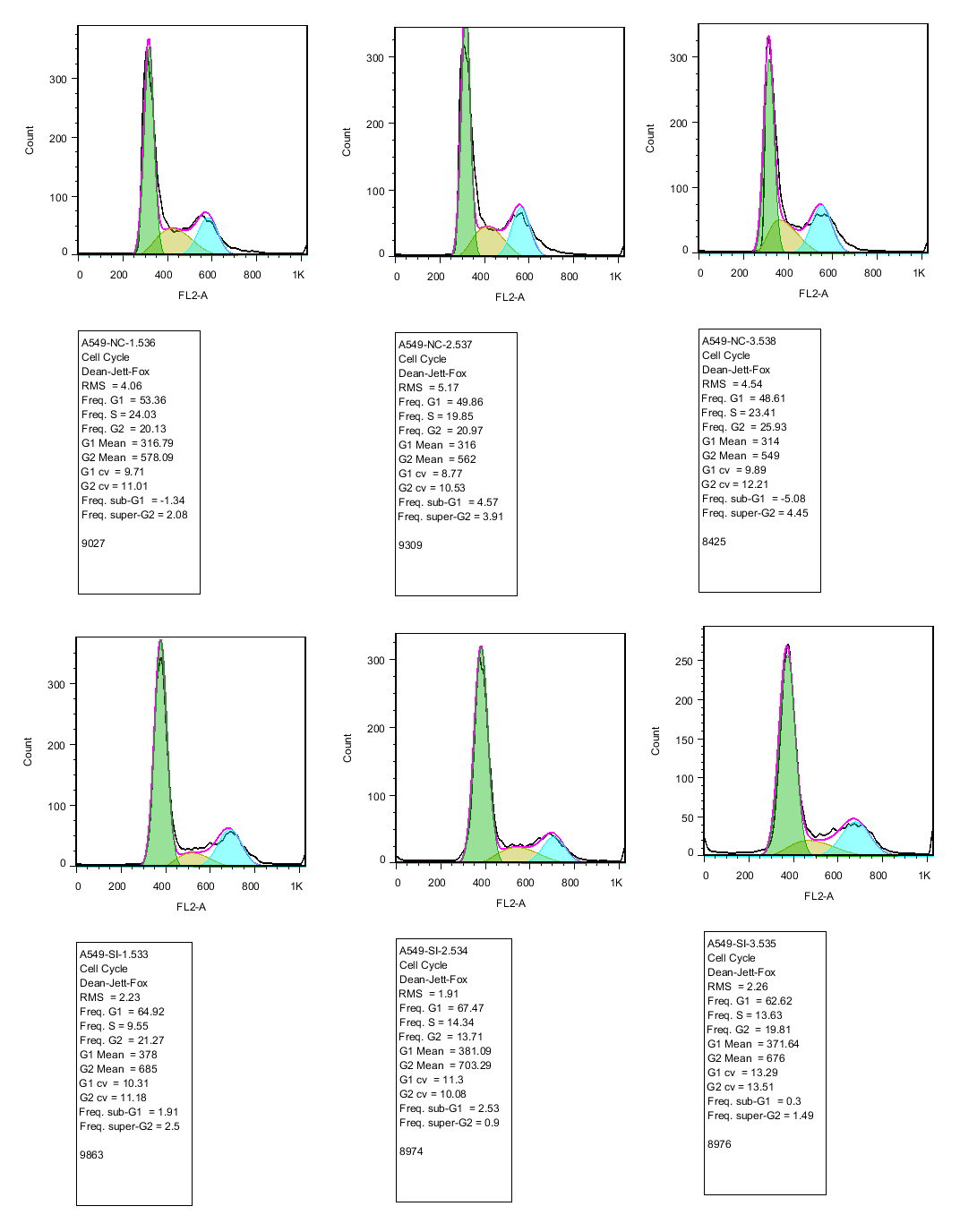

Supplement: Supplementary file 3 — Additional file 3. [file 12885_2023_11233_MOESM3_ESM.zip › A549.png]

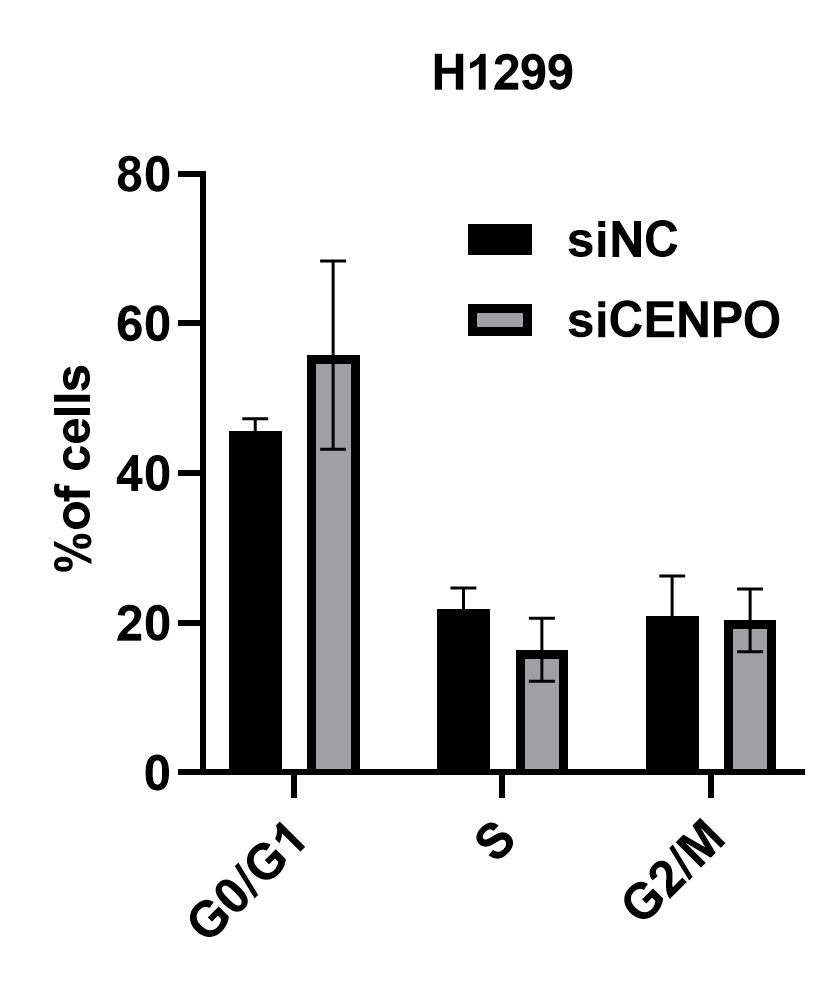

Supplement: Supplementary file 3 — Additional file 3. [file 12885_2023_11233_MOESM3_ESM.zip › H1299 Cell cycle.tif]

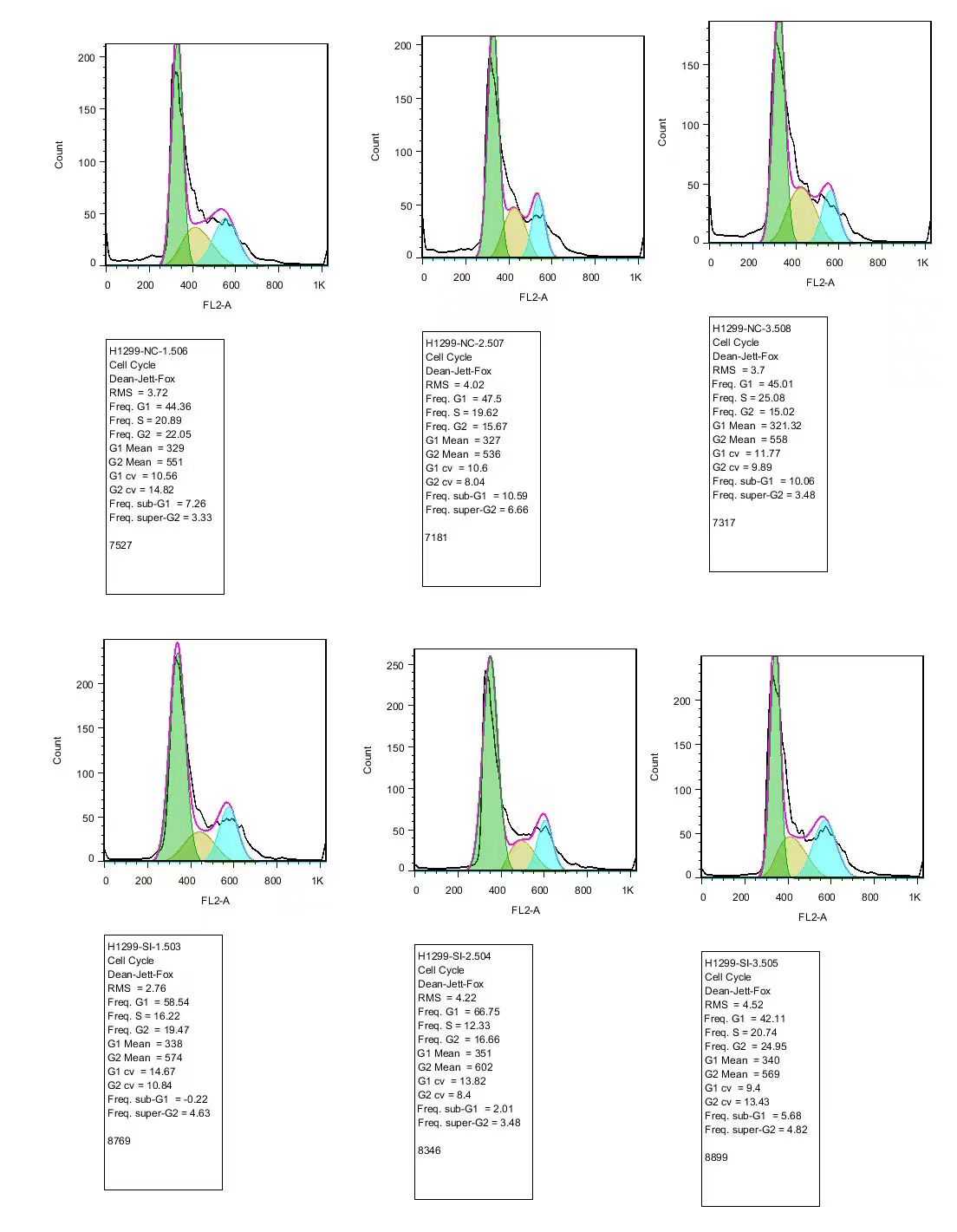

Supplement: Supplementary file 3 — Additional file 3. [file 12885_2023_11233_MOESM3_ESM.zip › H1299.jpg]

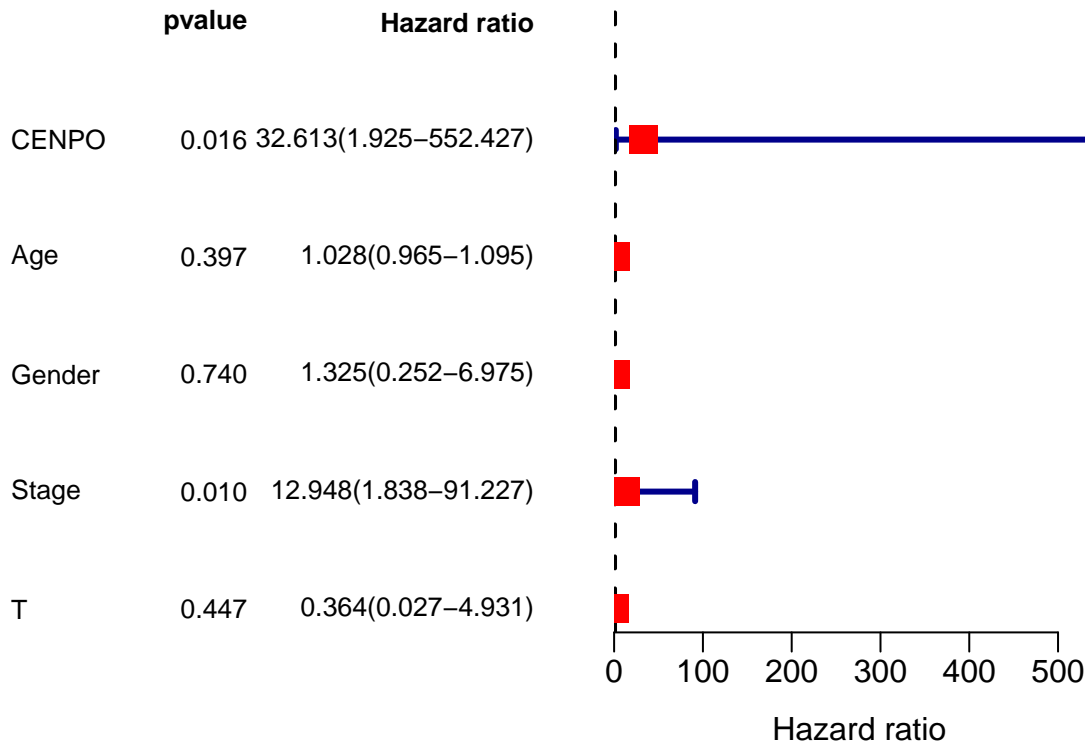

Supplement: Supplementary file 4 — Additional file 4. [file 12885_2023_11233_MOESM4_ESM.zip › CENPO-pan-cox/KICH-COX/11.cox/multiForest.pdf]

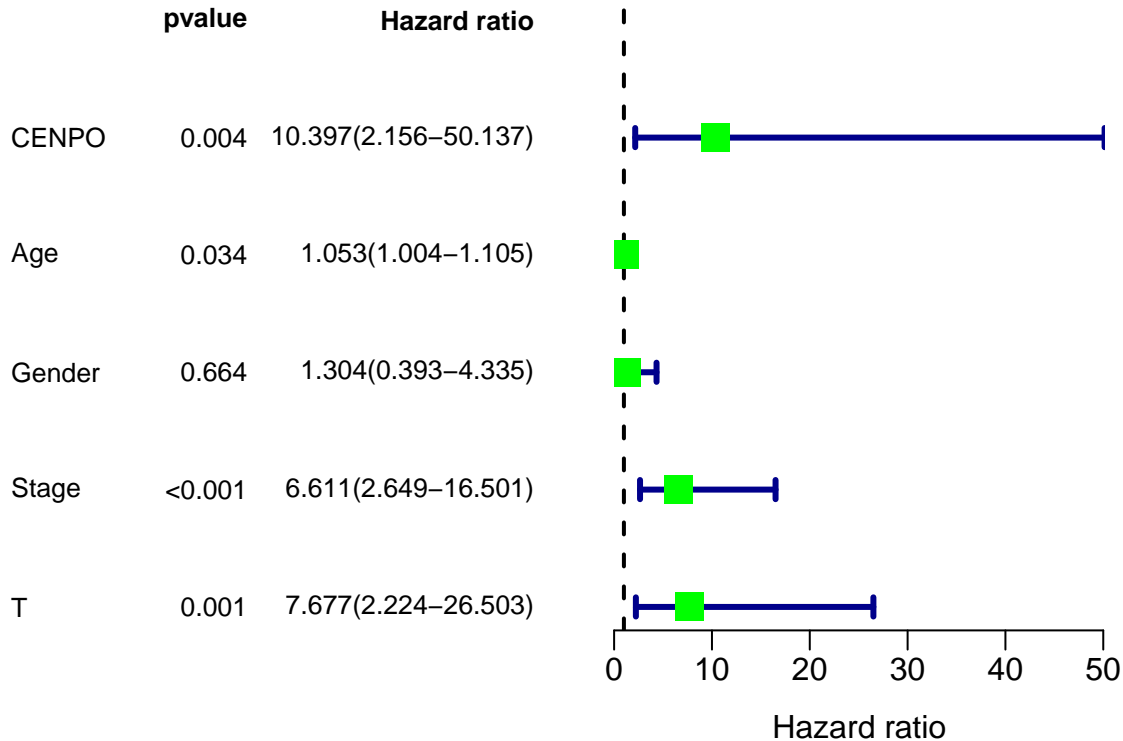

Supplement: Supplementary file 4 — Additional file 4. [file 12885_2023_11233_MOESM4_ESM.zip › CENPO-pan-cox/KICH-COX/11.cox/uniForest.pdf]

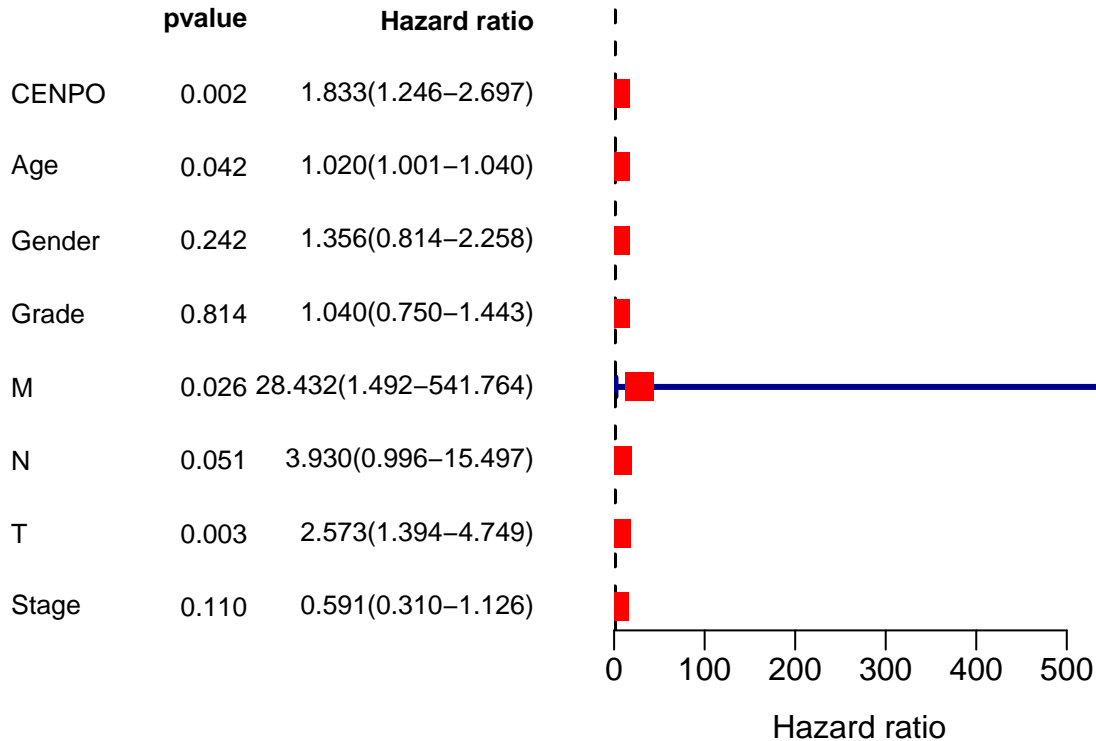

Supplement: Supplementary file 4 — Additional file 4. [file 12885_2023_11233_MOESM4_ESM.zip › CENPO-pan-cox/LIHC-COX/11.cox/multiForest.pdf]

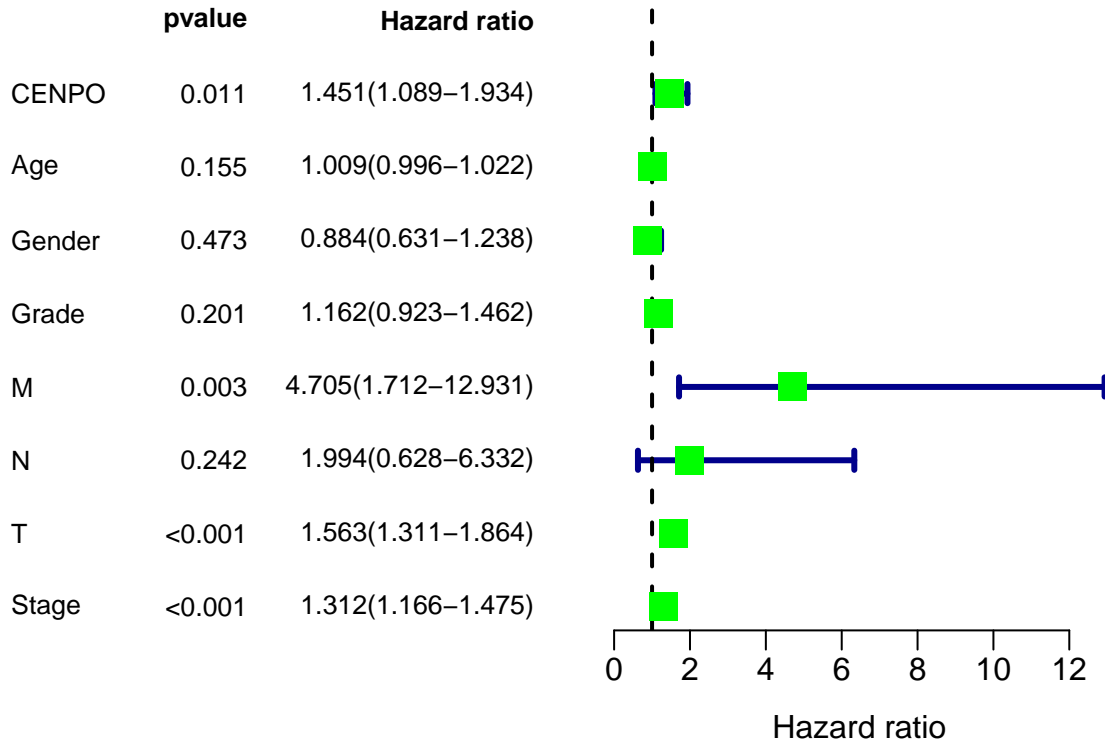

Supplement: Supplementary file 4 — Additional file 4. [file 12885_2023_11233_MOESM4_ESM.zip › CENPO-pan-cox/LIHC-COX/11.cox/uniForest.pdf]

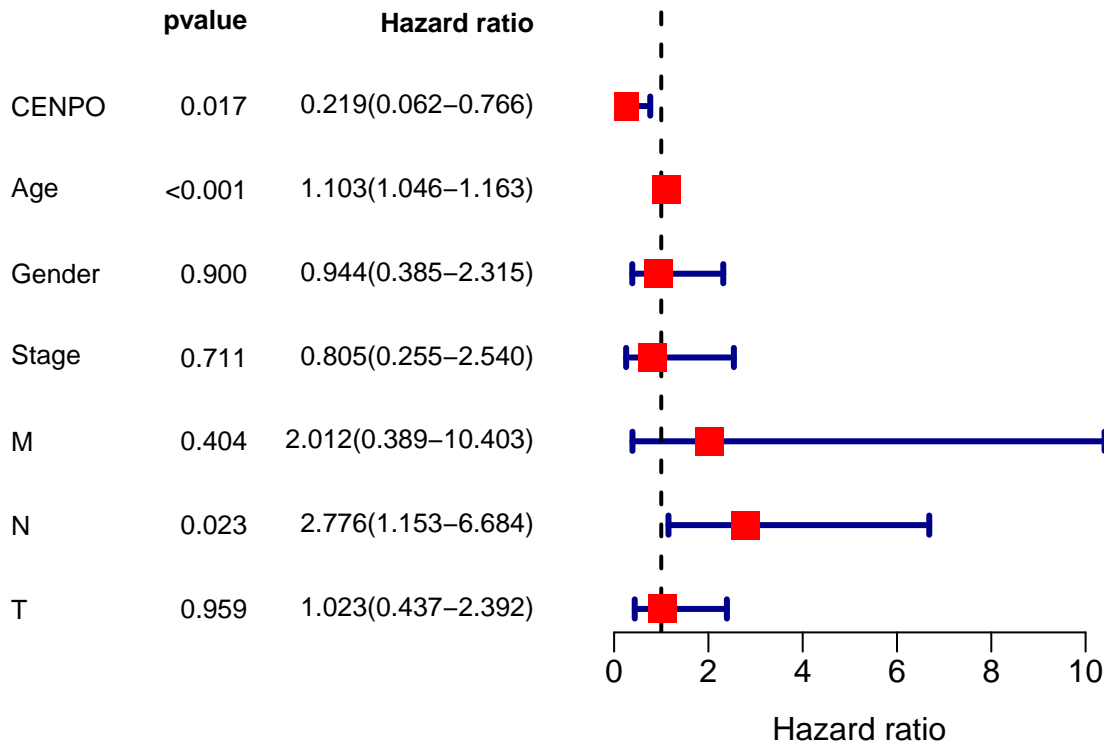

Supplement: Supplementary file 4 — Additional file 4. [file 12885_2023_11233_MOESM4_ESM.zip › CENPO-pan-cox/READ-COX/11.cox/multiForest.pdf]

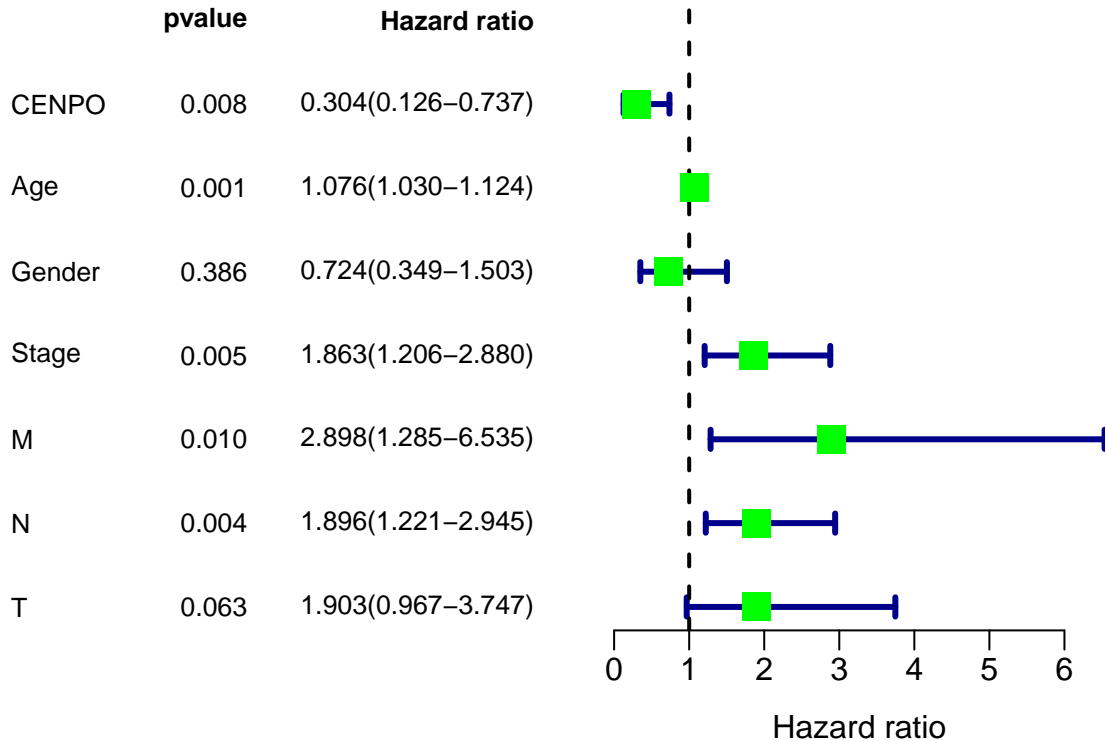

Supplement: Supplementary file 4 — Additional file 4. [file 12885_2023_11233_MOESM4_ESM.zip › CENPO-pan-cox/READ-COX/11.cox/uniForest.pdf]

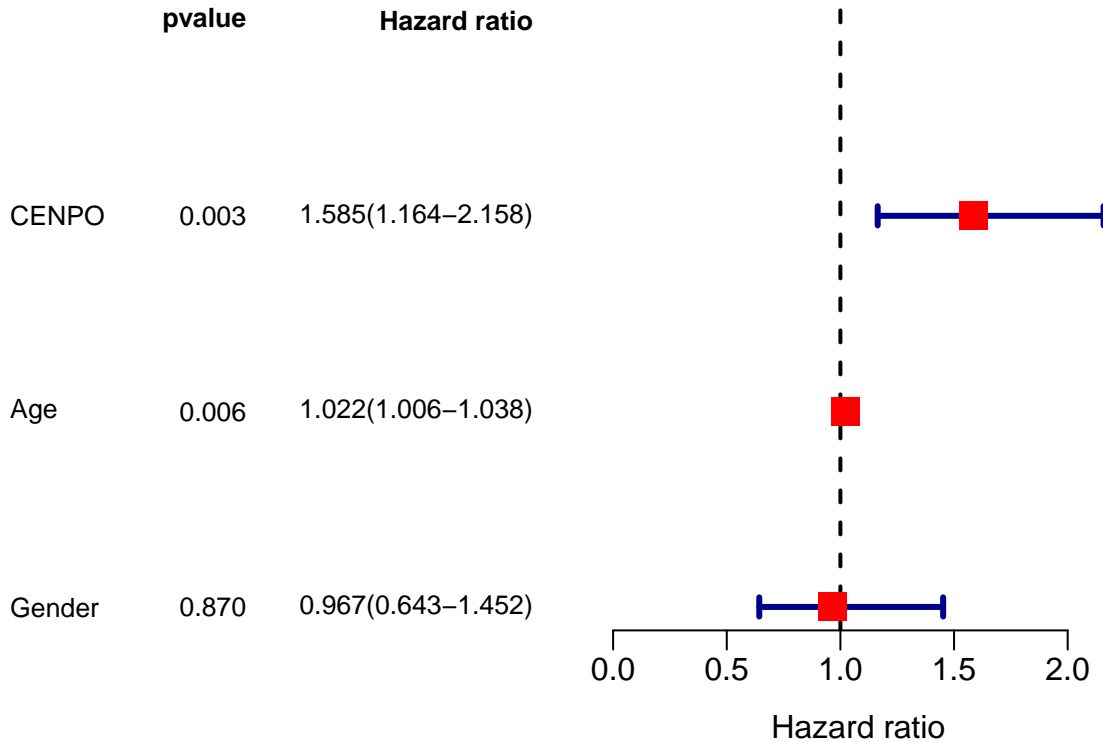

Supplement: Supplementary file 4 — Additional file 4. [file 12885_2023_11233_MOESM4_ESM.zip › CENPO-pan-cox/SARC-COX/11.cox/multiForest.pdf]

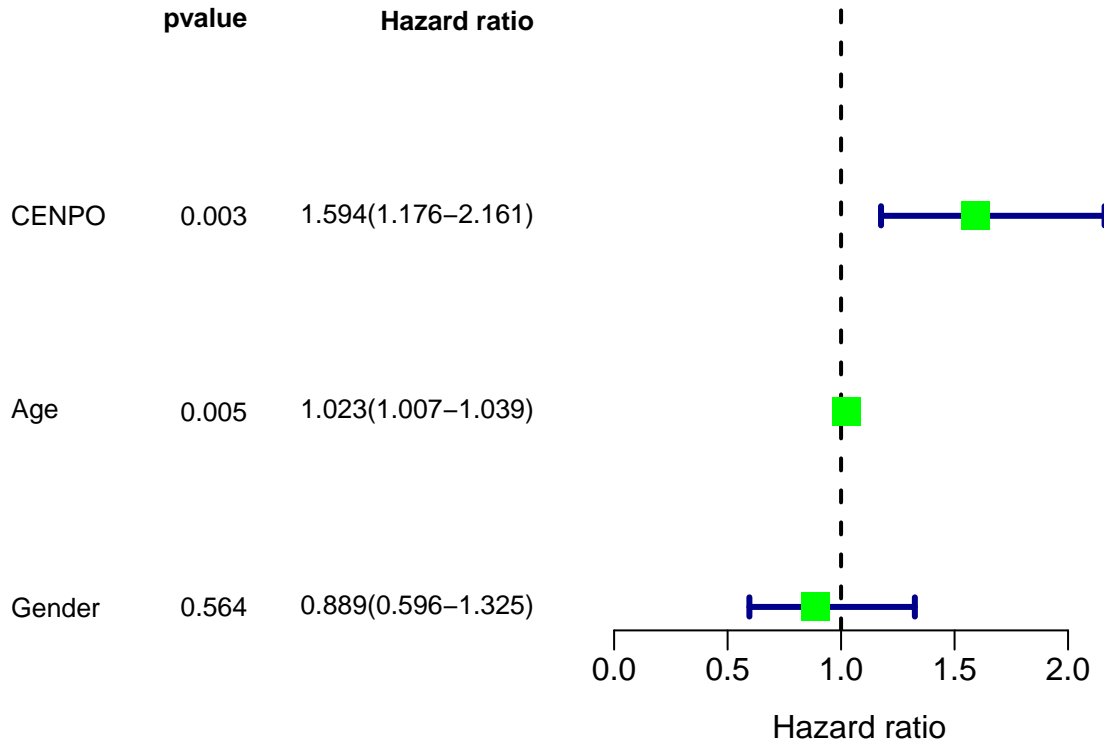

Supplement: Supplementary file 4 — Additional file 4. [file 12885_2023_11233_MOESM4_ESM.zip › CENPO-pan-cox/SARC-COX/11.cox/uniForest.pdf]

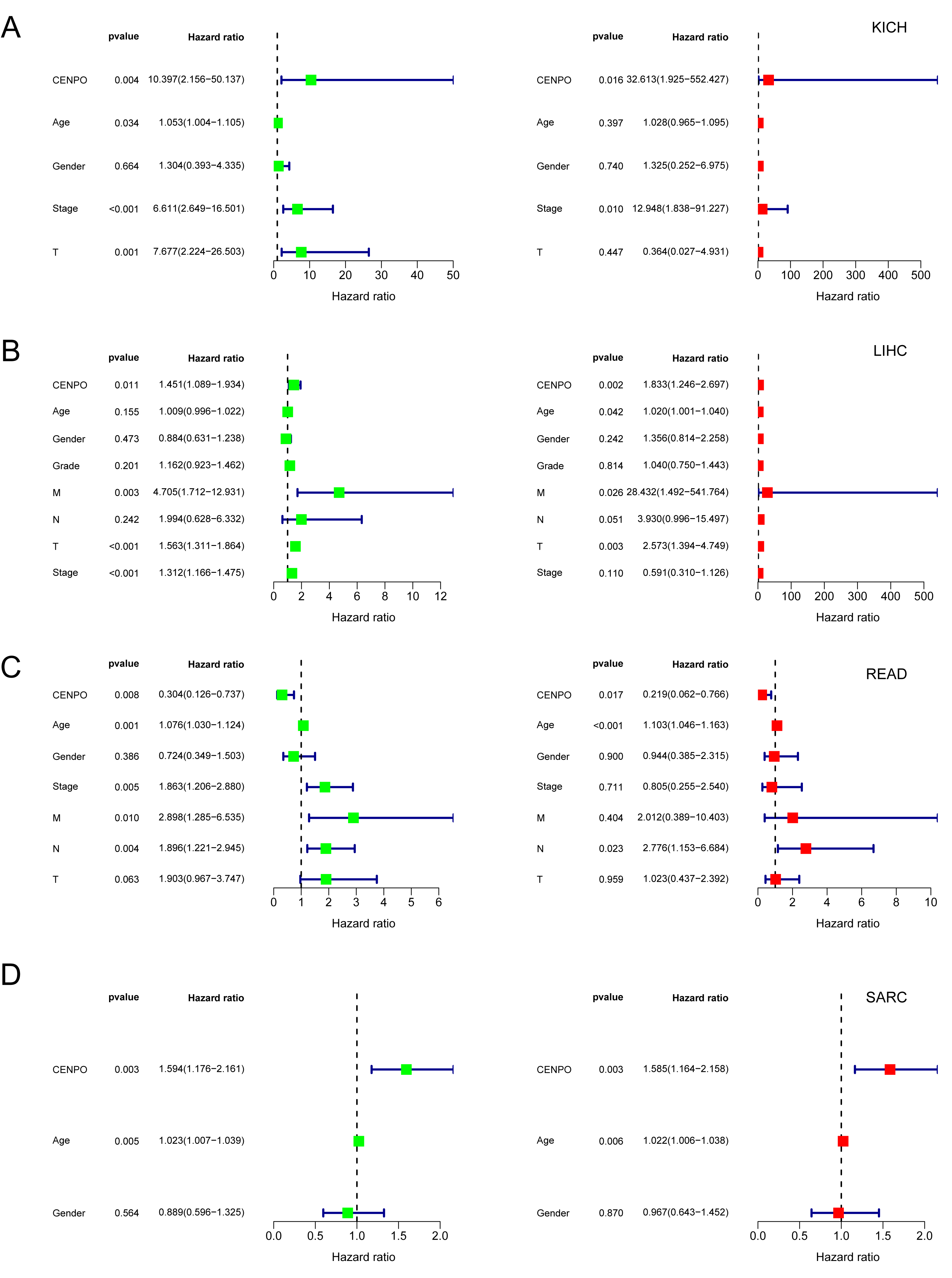

Supplement: Supplementary file 7 — Additional file 7. [file 12885_2023_11233_MOESM7_ESM.tif]

Repeat 1

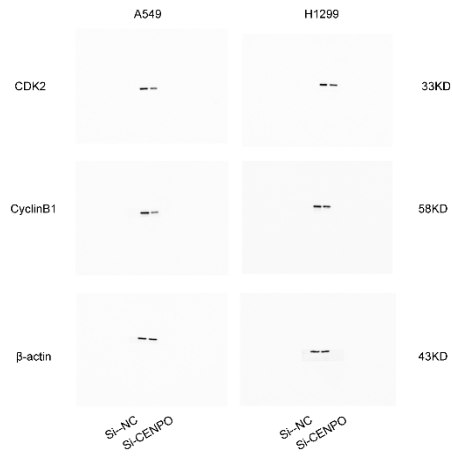

Repeat 2

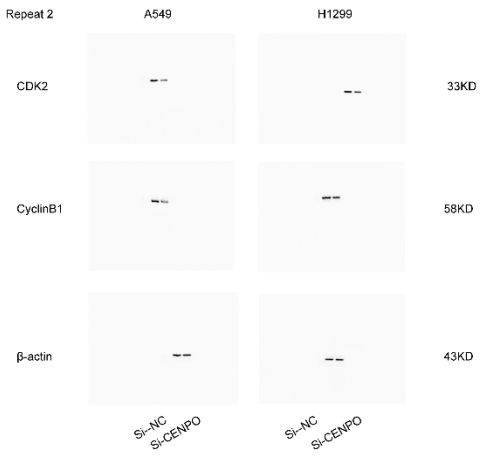

Repeat 3

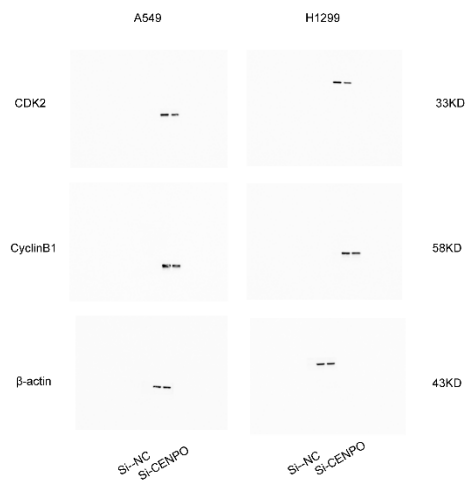

Supplement: Supplementary file 15 — Additional file 15. [file 12885_2023_11233_MOESM15_ESM.pdf]

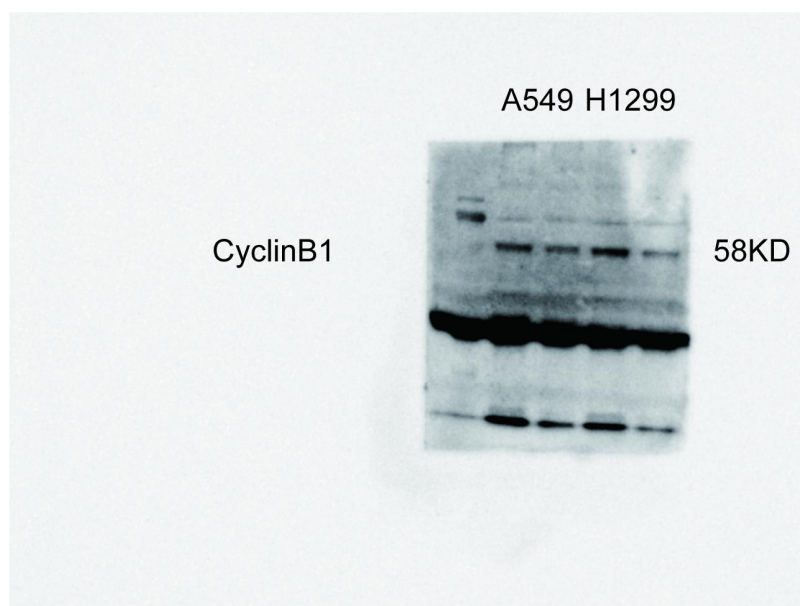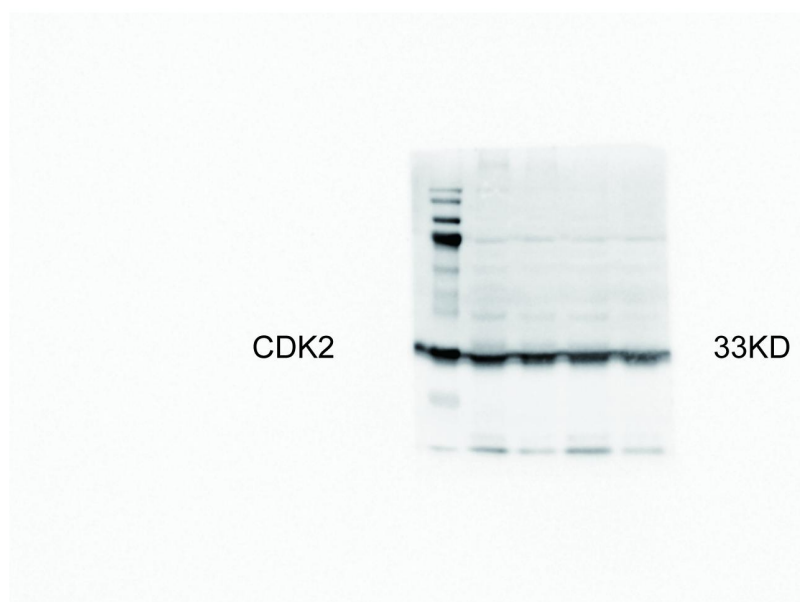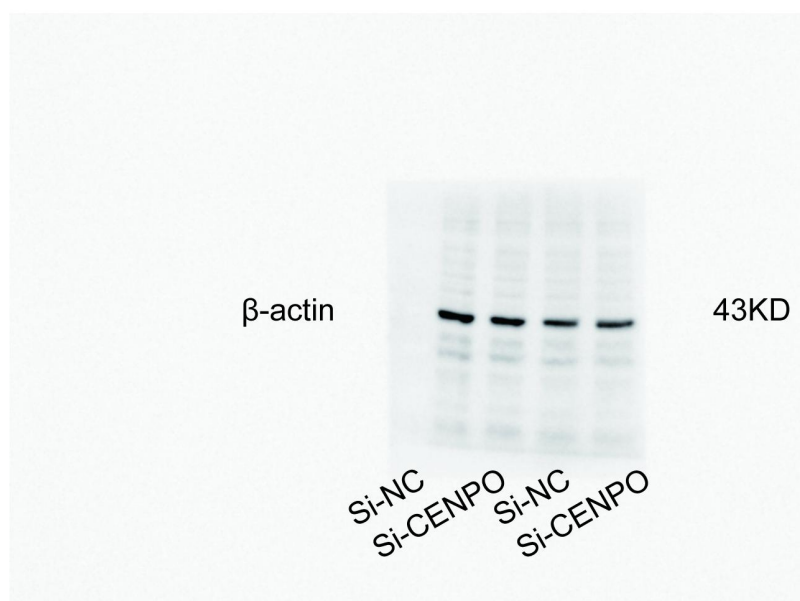

Supplement: Supplementary file 16 — Additional file 16. [file 12885_2023_11233_MOESM16_ESM.pdf]
